# A Computer-aided Study on the Tissue Changes in Oral Keratoses and Lichen Planus, and an Analysis of Case Groupings by Subjective and Objective Criteria

**DOI:** 10.1038/bjc.1970.49

**Published:** 1970-09

**Authors:** I. R. H. Kramer, R. B. Lucas, N. El-Labban, L. Lister

## Abstract

In a retrospective survey of 235 cases in which the diagnosis on biopsy was lichen planus, keratosis or leukoplakia, the histological features were re-assessed in as objective a manner as possible. For each case, the tissue changes were recorded under 37 headings, without any attempt at interpretation. The information was then transferred to punched cards, and used for various types of computer-aided analysis. Firstly, the frequency with which each tissue change occurred was assessed for each diagnostic group, and the data presented also show how often each change occurred in those cases that subsequently developed a carcinoma.

Re-analysis of the cases on the basis of short lists of tissue changes that were thought to characterize each diagnostic category showed, as expected, that many other factors must have played a part in the original diagnosis.

Analysis of the histological findings on an objective basis, using a cluster analysis programme, provided an encouraging degree of separation into the diagnostic groups. When a number of known carcinoma cases were included in the cluster analysis as “markers”, a small number of leukoplakia and keratosis cases were placed by the computer into the same cluster as these “markers”. Of the original 187 cases originally diagnosed as leukoplakia or keratosis, 4.8% are known to have developed a carcinoma, but of the 11 cases the computer placed in the same cluster as the “marker” cases of carcinoma, 36% have subsequently developed carcinoma. Thus, in the cluster analyses, the computer is tending to “recognize” those cases that later developed carcinoma, and to separate them from the bulk of the cases in which malignant change has not occurred.


### BRITISH JOURNAL OF CANCER

VOL. XXIV SEPTEMBER, 1970 NO. 3

### A COMPUTER-AIDED STUDY ON THE TISSUE CHANGES IN ORAL KERATOSES AND LICHEN PLANUS, AND AN ANALYSIS OF CASE GROUPINGS BY SUBJECTIVE AND OBJECTIVE CRITERIA

I. R. H. KRAMER, R. B. LUCAS, N. EL-LABBAN AND L. LISTER

*From the Departments of Pathology, Institute of Dental Surgery and Royal Dental Hospital of London School of Dental Surgery, University of London*

Received for publication May 5, 1970

**SUMMARY.—In a retrospective survey of 235 cases in which the diagnosis on biopsy was lichen planus, keratosis or leukoplakia, the histological features were re-assessed in as objective a manner as possible. For each case, the tissue changes were recorded under 37 headings, without any attempt at interpretation. The information was then transferred to punched cards, and used for various types of computer-aided analysis. Firstly, the frequency with which each tissue change occurred was assessed for each diagnostic group, and the data presented also show how often each change occurred in those cases that subsequently developed a carcinoma.**

**Re-analysis of the cases on the basis of short lists of tissue changes that were thought to characterize each diagnostic category showed, as expected, that many other factors must have played a part in the original diagnosis.**

**Analysis of the histological findings on an objective basis, using a cluster analysis programme, provided an encouraging degree of separation into the diagnostic groups. When a number of known carcinoma cases were included in the cluster analysis as " markers ", a small number of leukoplakia and keratosis cases were placed by the computer into the same cluster as these " markers ". Of the original 187 cases originally diagnosed as leukoplakia or keratosis, 4·8% are known to have developed a carcinoma, but of the 11 cases the computer placed in the same cluster as the " marker " cases of carcinoma, 36% have subsequently developed carcinoma. Thus, in the cluster analyses, the computer is tending to " recognize " those cases that later developed carcinoma, and to separate them from the bulk of the cases in which malignant change has not occurred.**

IT is well known that carcinoma of the mouth sometimes develops in an area where the mucosa is thickened, rough and whitish in appearance, and that this abnormality of the mucosa may have been present for a long time before the appearance of the cancer. Indeed, the relationship has so impressed clinicians and pathologists that the term " leukoplakia " traditionally has sinister connotations, and in the vast literature on this topic there are many estimates of the frequency of malignant change in such lesions. These vary from over 36% to below 2% (Pindborg *et al.*, 1968) with the more recent reports giving, in general, the lower figures. There are probably many reasons for this changing frequency; perhaps the most important is that syphilitic glossitis, so often accompanied by carcinoma, is now rarely seen. Furthermore, other clinico-pathological entities that give rise to white patches on the oral mucosa have been recognized in recent years, and very often these lesions appear not to be associated with the subsequent development of cancer. Nevertheless, even if only 4 to 5% of white patches on the oral mucous membrane become carcinomatous, it would certainly be of great importance if we could identify the lesions that would, in time, change in this way. Of course, certain changes in epithelium are well known as being precancerous, but even when such changes are seen there is no way of telling whether cancer will in fact develop, and when.

Because of these diagnostic and prognostic difficulties, a detailed study was undertaken of the histological features in a series of 235 cases. This study was retrospective, and therefore information is now available about the progress of most of the patients, although in many cases the natural progress of the lesions will have been altered by treatment.

The histological findings were submitted to several types of computer analyses, the objective being to see whether our diagnostic methods could be improved and, in particular, to see whether it was possible to identify more accurately the cases which were most likely to become malignant.

In this paper we present data on three analyses, concerning:

A. The frequency with which various tissue changes occurred in each of our diagnostic groups.
B. The effects of grouping by subjective criteria.
C. The effects of grouping by objective criteria, using computer-aided cluster analyses.

Preliminary accounts of these studies have been given elsewhere (Kramer *et al.*, 1969; Kramer, 1969).

The present study was based on cases that we had diagnosed as lichen planus, keratosis, or leukoplakia.

" Leukoplakia " is a term which, literally, describes any type of white patch on the mucosa, and the term is used by some in this simple descriptive sense. Others limit the use of the term to white lesions that cannot be assigned to any other diagnostic category (such as white sponge naevus or lichen planus), and the term has also been used as a histological diagnosis, especially for lesions judged to be precancerous.

It is now our practice to avoid " leukoplakia " as a histological term. However, during most of the period in which the biopsies in this retrospective survey were received, we reported " keratosis " for those lesions in which the tissue changes were less severe, and " leukoplakia " for the cases in which the changes were more marked and in which we wished to indicate that the lesion should be regarded as potentially precancerous. Whilst this distinction may not be valid, the separate diagnostic categories have been preserved in the presentation of our findings so that the validity of the distinction can be explored.

### MATERIALS AND METHODS

### *Patients*

The cases studied were those from which biopsies were received in the Departments of Pathology of the Royal Dental Hospital and the Eastman Dental Hospital, London, between the years 1952 to 1967, and which had been diagnosed as lichen planus, keratosis, or leukoplakia.

The mean ages of the patients, in years, at the time of biopsy were: keratosis, 49·4: leukoplakia 52·9: and lichen planus 47·5. The male–female ratios were keratosis 71 : 56, leukoplakia 40 : 20, and lichen planus 25 : 23.

Some patients had lesions confined to a single site, whilst in other patients the lesions were multiple or were so extensive as to involve multiple sites (only intraoral lesions are considered here). The ratios of single to multiple site lesions were keratosis 61 : 66, leukoplakia 26 : 34, and lichen planus 4 : 44.

In each group we considered by whom the lesions were first noticed (patient, dentist or doctor) with the results shown in Table I, and the reasons why the lesions were first noted (Table II).

The number of patients in whom a carcinoma developed must clearly be considered in relation to the period of follow-up; Table III shows the interval between

TABLE I.—*Who First Noticed the Lesions*

All figures are given as percentages of the cases in each diagnostic group

| | Keratosis | Leukoplakia | Lichen planus |
|---|---|---|---|
| Patient | 61 | 60 | 81 |
| Dentist | 39 | 38 | 19 |
| Doctor | 0 | 2 | 0 |

TABLE II.—*Why the Lesion was First Noted*

All figures are given as percentages of the cases in each diagnostic group. It will be seen that the total in each column exceeds 100%. This is because, in many cases, a combination of features led to the discovery of the lesion and it was not possible to be certain which was the first feature.

| | Keratosis | Leukoplakia | Lichen planus |
|---|---|---|---|
| Appearance | 79 | 73 | 56 |
| Pain | 23 | 30 | 50 |
| Other | 16 | 22 | 29 |

TABLE III

The number of cases in each period of " possible follow-up " related to the mean period of actual follow-up. The " possible follow-up " period is the period elapsed since biopsy and up to the date when the data were prepared for analysis.

| | Keratosis | | Leukoplakia | | Lichen planus | |
|---|---|---|---|---|---|---|
| Time since biopsy | No. of cases | Mean follow-up | No. of cases | Mean follow-up | No. of cases | Mean follow-up |
| < 1 | 4 | — | 1 | — | 3 | — |
| 1 | 11 | — | 9 | — | 5 | — |
| 2 | 20 | 1·3 | 7 | 1·7 | 11 | 1·1 |
| 3 | 26 | 2·2 | 4 | 1·3 | 5 | 0·8 |
| 4 | 18 | 3·2 | 8 | 3·4 | 3 | 3·3 |
| 5 | 9 | 4·1 | 8 | 4·4 | 1 | 5 |
| 6 | 14 | 4·7 | 9 | 5·2 | 3 | 5·3 |
| 7 | 5 | 3 | 3 | 4·7 | 1 | 7 |
| 8 | 6 | 7 | 5 | 4 | 1 | 8 |
| 9 | 2 | 8·5 | 1 | 8 | 6 | 8·8 |
| 10 | 5 | 6·6 | — | — | 2 | 10 |
| > 10 | 7 | 11·1 | 5 | 10 | 7 | 10·4 |

biopsy and the time when the data were prepared for the computer (*i.e.* the possible follow-up period) and the mean period of actual follow-up. For example, it will be seen that, of the cases diagnosed as keratosis, 18 were biopsied 4 years ago, and for these 18 cases the mean period of actual follow-up was 3·2 years.

### *Recording the Histological Features*

In all cases the histological diagnosis was originally made, or subsequently checked, by one or other of the two senior authors. No diagnosis was revised during this part of the study, as one of the purposes of the study was to compare the original diagnosis with the results of the computer-assisted analysis.

For each patient, a detailed form was completed which provided for the recording of personal data, the clinical features of the disease, and other factors such as smoking habits and the wearing of dentures. The present survey, however, relates only to the analysis of the histological findings, so the clinical data records will not be described here. Brief details of some of the clinical aspects are included to give an indication of the type of material studied.

New sections were prepared from each block, and serial sections were stained with haematoxylin and eosin, periodic acid Schiff (PAS) and PAS preceded by digestion with diastase. It was the intention that the histological study should be based on the type of material routinely used for reporting on such cases, so the main assessment was carried out on the sections stained with haematoxylin and eosin. However, the PAS preparations were included because of previous suggestions that the glycogen content of the epithelium, and changes in the basement membrane, may be of diagnostic importance in mucosal lesions of the type being studied (Cahn, Eisenbud and Blake, 1961, 1962; Komori, Welton and Kelln, 1966: Doyle, Manhold and Weisinger, 1968).

The purpose of the assessment was to provide a detailed record of the tissue changes, prepared in as objective a manner as possible, without any attempt at interpretation. When the assessments were being made, the individual recording the microscopic features of the section did not know the original diagnosis, or any of the clinical findings. The only clinical information available was the site from which the biopsy was taken, and this information was necessary because the structure of the normal mucosa varies from site to site within the mouth.

Before the assessments were started, each of the histological features to be recorded was specified as precisely as possible, and the two observers concerned examined trial groups of sections. These preliminary trials resulted in modifications in the specifications, and in the forms on which the records were to be made.

The process of examination of trial groups of sections, and modification of the specifications, was repeated until the differences between the two observers were reduced to what was considered to be an irreducible minimum. Thereafter, all assessments were made by these two observers.

The following were the tissue features recorded, together with some comments on the manner in which the assessments were made.

*Keratinization.*—This was recorded as normal, deficient, hyperorthokeratosis, parakeratosis or hyperparakeratosis.

In many cases an entry was made under more than one of the headings in this group; thus, if part of the lesion showed hyperorthokeratosis and part showed parakeratosis, both were recorded. For a biopsy from a site such as cheek mucosa, which is normally non-keratinized, absence of keratinization would be recorded as " normal ".

Hyperorthokeratosis was recorded when this layer was thicker than normal (with biopsies from sites in which keratinization is normally found) or where such a layer was present (however thin) in biopsies from areas that normally are not keratinized.

Similarly, a parakeratinized layer, however thin, was recorded as hyperparakeratosis if the biopsy came from a site where normally there is no keratinization.

*Intraepithelial keratinization.*—Cells in the stratum spinosum that were markedly more eosinophilic than their neighbours were regarded as showing keratinization. Many of these cells had a glassy cytoplasm and a pyknotic nucleus.

*Organisms in epithelium.*—These were recorded under two headings; hyphae and bacteria. Candida hyphae could usually be seen in the sections stained with haematoxylin and eosin, but the PAS sections were also used for assessing the presence of organisms in the epithelium.

*Vacuolization.*—This term is used to describe an appearance in the cells of the superficial part of the stratum spinosum. In these cells the greater part of the cytoplasm appears empty or unstained, although the nucleus is not displaced and there may be a peripheral cytoplasmic condensation. This change should be distinguished from hydropic degeneration, in which there are discrete spaces in the cytoplasm and the nucleus may be displaced.

*Acanthosis.*—Recognized by an increase in the thickness of the epithelium, or in the width of the epithelial processes, we recorded whether the acanthosis affected less than ½, about ½, or more than ½ of the lesional area shown in the biopsy. Adjacent normal mucosa included in the biopsy was disregarded.

*Spongiosis.*—The change was recorded as absent, slight, moderate or marked.

*Hydropic degeneration.*—This was recorded separately for the stratum basale and the stratum spinosum, and for each layer the change was recorded as absent, slight, moderate or marked.

*Inflammatory cells in epithelium.*—If there were inflammatory cells infiltrating the epithelium, we recorded whether they were acute (polymorphs) or chronic (lymphocytes and plasma cells). In an attempt to quantitate the intensity of the infiltration, we selected the area in which the inflammatory cells in the epithelium were most numerous, and noted whether a high-power field contained less than 5, less than 10, or more than 10.

*Cellular pleomorphism, nuclear hyperchromatism, and disturbance in the polarity of the epithelial cells.*—Each of these features was recorded as absent, slight, moderate or marked.

*Prominent nucleoli.*—Enlarged or abnormally prominent nucleoli were looked for in the stratum basale and the stratum spinosum. For each layer, the findings were recorded on a simple absent/present basis.

*Increased numbers of mitoses.*—Another part of this study included mitotic counts. However, in his routine work the pathologist who reports " increased mitotic activity " usually does so on a subjective basis, without making a count. Therefore, we thought it would be of interest to include such a subjective assessment in our survey, so that this assessment could later be compared with the actual count made by an independent observer. Increased mitotic activity was therefore recorded on a subjective yes/no basis, the stratum basale and the stratum spinosum being assessed separately.

*Mitoses of abnormal form.*—This too was recorded on a yes/no basis, the stratum basale and the stratum spinosum being considered separately.

*Liquefaction degeneration.*—This is a generally used term for which there are notable differences in definition, and many authors have not defined the term at all, presumably because they believed that it was always used in the same way. We have defined liquefaction degeneration as a change in the basal cell layer characterized by an eosinophilia of the basal cells that may be associated with a loss of demonstrable nuclear material.

*Separation.*—In some biopsies the epithelium has a tendency to separate slightly from the lamina propria, and this separation appears to have occurred either during excision or during processing and sectioning of the tissue. This separation is sometimes associated with liquefaction degeneration, and although in most cases it is essentially artifactual it probably indicates a loosening of the dermo-epidermal attachment.

*Distribution and density of inflammatory cell infiltration in the lamina propria.*—In this assessment, the connective tissue was considered in two zones; the upper or papillary layer, and the lower or reticular layer. For each zone, the intensity of inflammatory cell infiltration was recorded in five grades, ranging from absent to very dense.

*Nature of the inflammatory cells.*—The nature of the inflammatory cells in the two connective tissue zones was studied. In the analysis reported here, only lymphocytes and plasma cells are considered; in every case, these formed the great majority of the inflammatory cells. For both lymphocytes and plasma cells four grades were established—" absent ", " present " (*i.e.* only in small numbers), " numerous " (*i.e.* present in larger numbers but not forming the majority of the inflammatory cells), and " predominating ". Taken in conjunction with the assessment of density of cellular infiltration, these data provided an adequate picture of the amount and character of the infiltration.

The presence of Russell bodies was also recorded.

*Glycogen in the epithelium.*—The PAS-stained sections, with diastase controls, were used to assess the amount of glycogen demonstrable in the epithelium. For this purpose, the epithelium was considered in four zones, upper (superficial), middle, suprabasal and basal. Each zone was assessed as showing one of four grades of diastase-sensitive PAS reactivity; none, slight, moderate or marked.

*Basement membrane.*—In many previous studies on skin and mucosal lesions of various types, reference is made to changes in the basement membrane. However, these studies were often carried out using only sections stained with haematoxylin and eosin, and we believe that the basement membrane cannot be accurately identified in sections stained in this way, except perhaps in those cases where there is gross thickening. Such references to the integrity or discontinuity of the basement membrane usually appear to be based more on the architecture of the dermo-epidermal junction than on the particular examination of a distinctive subepidermal structure. There are differing opinions on the structure and origin of the so-called basement membrane; for the purposes of the present study this was defined as the band of material, immediately subjacent to the basal epithelial cells, that is markedly more PAS-positive than the underlying connective tissue. In normal oral mucosa this forms a thin continuous line; a marked increase in the depth was recorded as a thickening, whilst in cases showing areas of discontinuity this was recorded as a deficiency. If both changes were present in different parts of the same specimen, both were recorded.

When all of the records were completed, they were checked for internal consistency, and the details for each patient were then transferred to punched cards. Throughout the recording, and during the transfer of the data to punched cards, each patient was identified by a code number. Only after the various analyses had been made were the code numbers compared with a master list to see the original diagnosis for each patient.

This survey was based on 48 cases in which the original histological diagnosis was lichen planus, and 187 cases in which the histological diagnosis was non-diagnostic keratosis or leukoplakia. It should be noted here that, although we are referring to the original histological diagnosis (as opposed to the initial diagnosis made by the clinician), in some cases the pathologist's diagnosis would inevitably have been influenced by the clinical information he had available.

### *Methods of Analysis*

A. *Frequency of tissue changes*

In the first analysis we determined the frequency with which each of the tissue changes occurred in each of our diagnostic groups, thus providing quantitative information that has not previously been available for the majority of these features.

B. *Grouping by subjective criteria*

If a pathologist is asked to describe the tissue features that caused him to make a particular diagnosis, he can give a number of specified items. However, in difficult cases, he may find it impossible to put into words exactly why he reached one diagnosis as opposed to another. This is because many of the elements of pathological diagnosis lie at the subconscious level—the pathologist's equivalent of the well-known " clinical instinct ".

Therefore, we thought it would be of interest to see whether we could specify, in simple terms, the tissue changes that led us to give certain diagnoses.

In our series, we had 48 cases in which we had diagnosed lichen planus. Therefore, we prepared a short list of the tissue changes that we believed were most important in leading us to that diagnosis. The changes we specified were:

1. Abnormal keratinization (of any type—hyperorthokeratosis, parakeratosis, hyperparakeratosis).
2. The presence of a stratum granulosum.
3. Liquefaction degeneration of the basal cell layer.
4. A tendency for the epithelium to separate from the lamina propria.
5. An inflammatory cell infiltration mainly confined to the superficial layer of the lamina propria.
6. This inflammatory cell infiltration must consist mainly of lymphocytes.

In relation to items 1 and 2, it must be remembered that most of the biopsies in this group came from the buccal mucosa, a site that normally is not keratinized and has no stratum granulosum.

The computer was programmed to search our 235 cases, find those in which all

6 of the specified " lichen planus features " were present, and analyse these according to the diagnoses that had actually been made. Then the remaining cases were searched to find those having any combination of 5 out of the 6 features, any combination of 4 out of 6, and any combination of 3 out of 6. Each group thus found was analysed according to diagnosis. In other words, we were determining to what extent these 6 histological features led us to give a particular diagnosis.

We then repeated this type of analysis by attempting to specify the histological features that, we believed, had led us to make a diagnosis of leukoplakia (as noted earlier, we used to use " leukoplakia " as a term for the non-diagnostic keratoses showing the more severe tissue changes).

For this analysis, we specified the following 9 features:

1. Abnormal keratinization of any type (hyperorthokeratosis, parakeratosis, hyperparakeratosis).
2. Acanthosis.
3. Cellular pleomorphism.
4. Nuclear hyperchromatism.
5. Disturbance in the polarity of the epithelial cells.
6. Inflammatory cell infiltration in the superficial part of the lamina propria.
7. Inflammatory cell infiltration in the deeper part of the lamina propria.
8. These infiltrations to include lymphocytes.
9. These infiltrations to include plasma cells.

As in the case of the " lichen planus features ", the computer was programmed to find the cases showing all 9 features, then any combination of 8 out of 9, any combination of 7 out of 9, and so on. Each group of cases thus found was subdivided into the diagnoses that had actually been given.

No specification was necessary for the keratosis group, as the nature of the material was such that all cases not diagnosed as lichen planus or as leukoplakia would automatically fall into that group.

As will be shown later, the results of these analyses showed that our chosen histological features did not accurately define our actual diagnostic groups.

This not-unexpected result led us to the next stage in the analysis, a non-subjective grouping of the cases.

C. *Grouping by objective criteria* (*cluster analysis*)

If a group of individuals is taken, and for each individual two parameters are measured (such as height and weight) the results can be plotted on a graph. It is possible that the graph might show, not only a relationship between the two parameters, but also an uneven distribution. In the example shown in Fig. 1 the individuals, when classified by height and weight, appear to fall into two well-defined groups or " clusters ", and this suggests that the sample studied may contain two different types of individual.

If the number of parameters to be studied is 40, not 2, it is impossible to depict the distribution of the cases graphically. However, the computer can make such complex comparisons, and can determine whether the individuals in the sample fall into " natural groups " on the basis of similarity between the individuals in one group and difference from the individuals of other groups. Thus, by an

FIG. 1.—When two variables (such as height and weight) are measured and correlated, the results may show that the individuals fall into groups or " clusters ", suggesting that the population examined contains individuals of different types.

entirely objective and mathematical process, the computer can examine the recorded histological features of a series of cases, and can determine whether they appear to fall into differing " natural groups ". We can then look at each group of cases formed by the computer, to see what diagnoses we had actually given on these cases.

If the information supplied to the computer contained all the information we used in making our subjective diagnoses, if there are real differences between our diagnostic categories, and if we had shown complete consistency in our methods of reaching a diagnosis, then the clusters formed by the computer might correspond to our subjective diagnostic groups.

For the cluster analyses we used the 235 cases previously referred to, and we added 13 cases of squamous cell carcinoma as " markers ". The purpose of this addition was to see how the computer would cluster the carcinoma cases in relation to some of the others that we suspected to be precancerous.

For each case, the computer was supplied with information on 41 histological variables. It should be noted that, for the carcinoma cases, we deliberately omitted the information that the sections showed invasion.

An explanation of the mathematical basis of the cluster analysis programme is given in the Appendix to this paper.

This programme provided for specification of the number of groups or clusters that were to be formed. Theoretically, if we had four different diagnostic groups formed by conventional diagnostic techniques, then the computer would provide the best separation of cases if it were programmed to form four clusters. However, this would involve the assumptions mentioned earlier (that the information supplied contained everything relevant, that our own diagnostic groups were real, and that we were always consistent). We thought it desirable to avoid these assumptions, and therefore the computer was programmed to form 2, 3, 4, 5, 6 and then 7 clusters.

In the output, the computer stated which cases it had placed in each cluster, so that we could analyse each cluster to see what diagnoses we had originally given.

RESULTS

In each table, " keratosis " and " leukoplakia " are given as separate diagnostic categories, for the reasons explained earlier.

### A. *Frequency of various tissue changes*

As described previously, many of the histological features were assessed on a roughly quantitative basis in up to four grades, and often the stratum spinosum and the stratum basale were assessed separately. This produced a large amount of data for the computer analyses, but it is necessary to condense the data for presentation here in tabulated form.

Therefore, where the quantitative assessments produced no marked differences, we reclassified the findings on a simple " present/absent " basis. In other instances, we have shown not only the frequency with which the change occurred, but also the effect of eliminating the lowest quantitative grade (*i.e.* the effect of raising the threshold). For example, for spongiosis, we show the percentage of cases in each diagnostic group showing any spongiosis, and then the percentage showing more than the least degree recognized (this is indicated in Tables IV to VII as $> +$).

Thus, Tables IV to VII give the percentage of cases in each diagnostic group showing the change indicated. In addition, we show (in the columns marked M) the proportion of cases showing the change, in which a carcinoma later developed.

Tables IV–VII give the *percentage* of cases in each diagnostic group showing each of the histological features. In addition, the columns headed "M" indicate what *proportion* of cases, showing each change, later developed a carcinoma.

TABLE IV

| | | Keratosis | | Leukoplakia | | Lichen planus |
|---|---|---|---|---|---|---|
| | | % | M | % | M | % |
| 1 | Keratin deficiency | 2 | — | 2 | — | 10 |
| 2 | Hyperorthokeratosis | 65 | 1/83 | 47 | 5/28 | 44 |
| 3 | Parakeratosis | 13 | — | 17 | 1/10 | 13 |
| 4 | Hyperparakeratosis | 47 | 1/60 | 53 | 4/32 | 52 |
| 5 | Vacuolization | 17 | 1/21 | 8 | — | 6 |
| 6 | Candida | 6 | 1/8 | 28 | 3/17 | 8 |
| 7 | Granular layer | 76 | 1/97 | 67 | 4/40 | 77 |
| 8 | Acanthosis | 88 | 2/112 | 95 | 7/57 | 52 |
| | > + | 71 | 2/91 | 88 | 6/53 | 38 |
| 9 | Atrophy | 6 | — | 8 | 1/6 | 42 |
| 10 | Ulceration | 2 | — | 12 | 3/7 | 8 |
| 11 | Liquefaction degen. | 12 | — | 17 | 2/10 | 90 |
| 12 | Separation | 13 | 1/17 | 30 | 2/18 | 65 |
| 13 | Spongiosis | 22 | 1/28 | 50 | 5/30 | 73 |
| | > + | 10 | — | 23 | 3/14 | 33 |
| 14 | Hydropic spinous | 55 | 2/70 | 65 | 5/39 | 90 |
| | > + | 15 | 1/19 | 30 | 4/18 | 40 |
| 15 | Hydropic basal | 40 | 2/51 | 55 | 4/33 | 92 |
| | > + | 6 | 1/8 | 15 | 1/9 | 54 |
| 16 | Acute inf. cells | 7 | — | 22 | 1/13 | 8 |
| 17 | Chronic inf. cells | 65 | 1/82 | 82 | 7/49 | 98 |
| | > + | 47 | 1/59 | 75 | 7/45 | 94 |

### TABLE V

| | | Keratosis | | Leukoplakia | | Lichen planus |
|---|---|---|---|---|---|---|
| | | % | M | % | M | % |
| 18 | Intra-ep. keratin | 22 | 1/28 | 35 | 5/21 | 4 |
| 19 | Pleomorphism | 23 | 1/29 | 58 | 6/35 | 42 |
| | > + | 6 | — | 32 | 4/19 | 4 |
| 20 | Hyperchromatism | 14 | — | 47 | 4/28 | 23 |
| | > + | 2 | — | 12 | 3/7 | 0 |
| 21 | Disturbed polarity | 20 | 1/25 | 53 | 7/32 | 25 |
| | > + | 2 | — | 23 | 3/14 | 0 |
| 22 | Enlarged nucleoli sp. | 27 | 1/34 | 53 | 6/32 | 67 |
| 23 | Enlarged nucleoli basal | 8 | — | 20 | 1/12 | 38 |
| 24 | Increased mitoses sp. | 7 | 1/9 | 25 | 1/15 | 2 |
| 25 | Increased mitoses basal | 4 | — | 17 | — | 2 |
| 26 | Abnormal mitoses sp. | 1 | — | 22 | 5/13 | 0 |
| 27 | Abnormal mitoses basal | 0 | — | 5 | 2/3 | 0 |

### TABLE VI

| | | Keratosis | | Leukoplakia | | Lichen planus |
|---|---|---|---|---|---|---|
| | | % | M | % | M | % |
| 28 | Inflam. cells. Upper | 72 | 1/92 | 95 | 7/57 | 100 |
| | > + | 30 | 1/38 | 70 | 5/42 | 94 |
| 29 | Inflam. cells. Lower | 22 | — | 45 | 5/27 | 54 |
| | > + | 7 | — | 32 | 2/19 | 35 |
| 30 | Lymphocytes | 74 | 1/94 | 95 | 7/57 | 100 |
| | > + | 36 | 1/47 | 70 | 7/42 | 100 |
| 31 | Plasma cells | 24 | 1/31 | 75 | 7/45 | 52 |
| | > + | 7 | 1/9 | 32 | 4/19 | 6 |
| 32 | Russell bodies | 7 | 1/9 | 40 | 5/24 | 17 |

### TABLE VII

| | | Keratosis | | Leukoplakia | | Lichen planus |
|---|---|---|---|---|---|---|
| | | % | M | % | M | % |
| 33 | P.A.S. Upper | 57 | 2/72 | 48 | 3/29 | 44 |
| | > + | 22 | — | 15 | 1/9 | 19 |
| 34 | P.A.S. Middle | 65 | 2/82 | 68 | 5/41 | 81 |
| | > + | 22 | — | 22 | 3/13 | 25 |
| 35 | P.A.S. Suprabasal | 7 | — | 2 | — | 8 |
| | > + | — | — | — | — | 2 |
| 36 | Basement mem. Thick | 36 | — | 48 | 3/29 | 71 |
| 37 | Basement mem. Def. | 12 | 1/15 | 35 | 3/21 | 46 |

### B. *Analyses by subjective criteria*

Tables VIII and IX show the results of analysing the cases according to our 6 stated " lichen planus features " and our 9 stated " leukoplakia features ".

### TABLE VIII

The percentage of cases in each diagnostic category related to the number of the " lichen planus characteristics " present.

| Original diagnosis | Number of features present | | | |
|---|---|---|---|---|
| | < 4 | 4 | 5 | 6 |
| Leukoplakia | 61 | 17 | 22 | 0 |
| Keratosis | 74 | 21 | 5 | 0 |
| Lichen planus | 0 | 31 | 25 | 44 |

TABLE IX

The percentage of cases in each diagnostic category related to the number of the "leukoplakia characteristics" present.

| Original diagnosis | Number of features present <3 | 3 | 4 | 5 | 6 | 7 | 8 | 9 |
|---|---|---|---|---|---|---|---|---|
| Leukoplakia | — | 1 | 12 | 17 | 15 | 15 | 25 | 15 |
| Keratosis | 20 | 8 | 24 | 25 | 12 | 8 | 3 | 0 |
| Lichen planus | 2 | 6 | 19 | 38 | 17 | 4 | 10 | 4 |

From Table VIII, it will be seen that, whenever all 6 of the "lichen planus features" were present, the case had actually been diagnosed as lichen planus. Also, whenever less than 4 of the 6 features were present, the case had been diagnosed as keratosis or leukoplakia, not lichen planus. However, when the cases showed any combination of 5 or 4 of the 6 features, they were sometimes diagnosed as lichen planus, sometimes as keratosis, and sometimes as leukoplakia.

Thus, in "classical" cases of lichen planus our 6 features defined the diagnosis, and in cases lacking more than 2 of the features, we never made that diagnosis. But when 5 or 4 features were present, our lichen planus cases overlapped those given another diagnosis. Of course, all of these analyses are based on the provisional assumption that our diagnoses were always correct. This is unlikely to be so, but there is usually no objective way of checking on this point: if a confident diagnosis could have been reached on other forms of evidence, the biopsies would not have been taken.

The analysis of the cases on the basis of the 9 features relating to "leukoplakia" (Table IX) showed an ever greater overlap between cases given this and other diagnoses. Clearly, these 9 features did not adequately define this diagnostic category.

C. *Analyses by objective criteria*

Tables X and XI show how the computer assigned the cases according to the number of clusters specified. In each cluster, we have shown the actual diagnoses that had been given on the cases in each cluster.

If each diagnostic category is taken in turn, and if we examine how many of these cases remained together in one cluster, we see that, with increasing numbers of clusters, smaller numbers of each diagnostic group remain together in any one cluster. For example, there were 127 cases diagnosed originally as "keratosis". When two clusters are formed, 123 of these 127 are placed together in one cluster, when three clusters are formed the number of keratosis cases grouped together falls to 120, and by the seven-cluster analysis only 84 cases of keratosis remain together.

TABLE X

The 248 biopsies divided into 2 and 3 clusters by the computer on the basis of the recorded histological features. Each cluster formed by the computer has been analysed to show the original histological diagnoses.

| Original diagnosis | Number of biopsies | Two-cluster analysis 1 | 2 | Three-cluster analysis 1 | 2 | 3 |
|---|---|---|---|---|---|---|
| Carcinoma | **13** | **13** | — | **13** | — | — |
| Leukoplakia | **60** | 17 | **43** | 19 | **34** | 7 |
| Keratosis | **127** | 4 | **123** | 3 | **120** | |
| Lichen planus | **48** | 4 | **44** | 1 | 18 | **29** |

TABLE XI

The same cases as in Table X, now divided by the computer into 4, 5, 6 or 7 clusters.

| Original diagnosis | Number of biopsies | Four-cluster analysis | | | | Five-cluster analysis | | | | |
|---|---|---|---|---|---|---|---|---|---|---|
| | | 1 | 2 | 3 | 4 | 1 | 2 | 3 | 4 | 5 |
| Carcinoma | **13** | **12** | — | — | 1 | **13** | — | — | — | — |
| Leukoplakia | **60** | 14 | **30** | 3 | 13 | 13 | **25** | 1 | 9 | 12 |
| Keratosis | **127** | 2 | **112** | 5 | 8 | 2 | **100** | 3 | 15 | 7 |
| Lichen planus | **48** | — | 6 | **40** | 2 | — | 6 | **33** | 7 | 2 |

| Original diagnosis | Number of biopsies | Six-cluster analysis | | | | | | Seven-cluster analysis | | | | | | |
|---|---|---|---|---|---|---|---|---|---|---|---|---|---|---|
| | | 1 | 2 | 3 | 4 | 5 | 6 | 1 | 2 | 3 | 4 | 5 | 6 | 7 |
| Carcinoma | **13** | **12** | — | — | 1 | — | — | **11** | — | — | — | — | — | 2 |
| Leukoplakia | **60** | 9 | **21** | 2 | 11 | 7 | 10 | 8 | 17 | — | 10 | 7 | 8 | 10 |
| Keratosis | **127** | 2 | **89** | 4 | 9 | 21 | 2 | 1 | **84** | 1 | 11 | 20 | 4 | 6 |
| Lichen planus | **48** | — | 2 | **34** | 2 | 7 | 3 | — | 1 | **33** | — | 7 | 3 | 4 |

However, if we examine the diagnostic purity of each cluster, a different picture emerges. In the two-cluster analysis, cluster 2 contains 123 cases of keratosis, but this cluster also contains 43 cases diagnosed as leukoplakia and 44 cases diagnosed as lichen planus. Thus, if a case is assigned by the computer to cluster 2, there is only a little more than an even chance that it was diagnosed as keratosis. However, if we move forward to the seven-cluster analysis, we now find that cluster 2 contains 84 cases diagnosed as keratosis, 17 cases diagnosed as leukoplakia (a diagnosis closely related to, and possibly only artificially distinguishable from keratosis) and no more than 1 case of lichen planus. Also in the seven-cluster analysis, we find that cluster 3 contains 33 cases diagnosed as lichen planus and only 1 case given another diagnosis.

Another interesting feature concerns the clustering of cases with those diagnosed as carcinoma. One of the most important purposes of these studies was to see whether the computer analyses would reveal anything of prognostic importance. As this was a retrospective survey, we had information on the subsequent progress of most of the patients. At the time that the data were finalized for computer analysis, we knew that, of the 60 cases originally diagnosed as leukoplakia, 7 later developed carcinoma, and of the 127 cases diagnosed as keratosis 2 later developed carcinoma.

If we examine the six-cluster analysis, it will be seen that most of the " marker " cases of carcinoma were placed in cluster 1. However, this cluster also contains 9 out of the 60 cases originally diagnosed as leukoplakia and 2 out of the 127 originally diagnosed as keratosis. Why did the computer place these 11 cases in the same cluster as the carcinomas? Out of the original 187 cases originally diagnosed as leukoplakia or keratosis, we now know that 4·8% have developed carcinoma. But, of the 11 cases that the computer separated from the others and placed in the same cluster as the marker cases of carcinoma, 36% have subsequently developed malignancy. Thus it is clear that, from the histological data supplied, the computer is tending to " recognize " those cases that subsequently developed malignancy, and to place them with the cluster marked by the known carcinoma cases.

### DISCUSSION

### (a) *Clinical data*

The mean ages of the patients differed little between the three different groups, and it is possible that the differences were unrelated to age of onset. Thus, it seems at least possible that some cases diagnosed as keratosis (mean age at biopsy 49·4 years) might have progressed to the more severe leukoplakia (mean age at biopsy 52·9 years). Conversely, the higher mean age of the leukoplakia cases might reflect the greater severity of the changes in cases of longer duration.

The lowest mean age was in the lichen planus cases (47·5 years), and this should be considered together with the reasons for discovery of the lesions as shown in Table II. This table shows that pain was much more common in lichen planus (a feature also noted by Levin (1957), Darling and Crabb (1954), Radden (1966) and others), and this might lead to earlier discovery and thus to earlier biopsy.

In the present series, keratosis and leukoplakia were more common in men, whilst in lichen planus there were almost equal numbers of males and females; this latter finding was similar to those of Radden (1966) and Darling and Crabb (1954) but differs from those of Cooke (1954) and Andreasen (1968) whose series showed a marked predominance of females.

In cases diagnosed as keratosis there were almost equal numbers with lesions at single and at multiple sites. In leukoplakia cases, there were more with multiple site lesions (single : multiple ratio 1 : 1·3), but with lichen planus almost all patients had involvement of more than one site (single : multiple ratio 1 : 11). This finding is typical of lichen planus, but the distribution of the keratosis and leukoplakia lesions is, at least in part, a reflection of the type of patient in this survey. Had we been dealing, for example, with syphilitic leukoplakias, many more patients would have had more extensive oral lesions.

Table I shows that, whereas 60% of the keratosis and leukoplakia lesions were first noticed by the patient, the corresponding figure for lichen planus was 81%. It seems likely that this higher incidence of discovery by the patient is related to the higher incidence of pain in the lichen planus group. It is of interest to note that almost all of the lesions not noticed by the patient were found by the dentist; only in one case were the lesions found by the doctor. However, these figures may be biased; if the dentist found the abnormality he would be likely to refer the patient to a dental hospital or to a large dental department, and most of our biopsies came from these sources, but if a doctor found the lesions the patient would more often be referred to a general hospital.

### (b) *Histological data*

In considering the histological data it must be remembered that the frequency with which a given change is found may be no more than a reflection of the importance that the pathologist attaches to the finding of that change in order to make the diagnosis. For example, a dense lymphocytic infiltration of the lamina propria is, in our view, an important feature in establishing the diagnosis of lichen planus. Therefore, an analysis of the cases we diagnosed as lichen planus will inevitably show that most have this features.

There is an extensive literature on the general histological features of oral keratosis, leukoplakia and lichen planus. However, in most previous publications the tissue changes are discussed without any detailed analysis or quantitative assessment of the frequency of individual features. Therefore, there is little previous work with which the results of the present study can be compared.

Most of our findings were in agreement with the usual descriptions of these disorders; this was to be expected, because we had assigned our cases to the various diagnostic categories on the basis of generally accepted criteria. However, the quantitative information that we obtained did reveal many interesting (and sometimes unexpected) results.

Tables IV to VII show the frequency with which each tissue change was found in each diagnostic category, thus providing quantitative information of a type that, for the most part, was not available previously.

However, it is rarely possible to make the most meaningful interpretation of any findings in isolation; it is necessary to correlate one change with another or with several others. For example, the glycogen content of the epithelium needs to be considered in relation not only to the diagnosis, but also to the type of keratinization and the degree of inflammation.

Many of these simple correlations have been made, together with more complex computer-aided analyses, and these will be presented elsewhere.

The principal importance of this group of mucosal lesions lies in the well-established finding that, in a number of cases, a squamous cell carcinoma will develop in the affected mucosa.

Therefore, one of our main objectives in the present study was to try to find better methods by which to identify those cases most likely to develop a carcinoma. As thls was a retrospective survey, we know which patients had developed a carcinoma subsequent to biopsy, and in Tables IV to VII we have shown how many cases, with each histological feature, later developed a carcinoma.

It should be emphasized that, in Tables IV to VII, the figure for the frequency of each histological feature is given as a percentage of all cases in that diagnostic group, whilst in the M (malignancy) columns the fraction figure indicates the *actual* number developing carcinoma and the *actual* number with the particular histological feature.

For example, reference to the first entry in Table V shows that intra-epithelial keratinization was seen in 22% of all cases diagnosed as keratosis. The next column shows that of these 28 cases, 1 developed a malignancy. Similarly, intra-epithelial keratinization was seen in 35% of all cases diagnosed as leukoplakia: of these 21 cases, 5 developed carcinoma. We have presented the information relating to the development of malignancy in this way, because the total number was small and we believe that it could be misleading to convert these findings to percentages.

The individual findings are often of interest: for example, only 7 cases of leukoplakia showed ulceration, and of these 3 became malignant. However, we present our findings without further comment, because in a subsequent paper we shall describe in detail the results of a computer-aided analysis that gave a quantitative assessment of the value of each tissue change in identifying those cases that subsequently developed a carcinoma.

Almost all methods of classification are based on the observation of similarities between individuals placed into one group, and dissimilarities between these individuals and those that are placed in other groups. Both clinical and histological diagnoses are based on this principle. However, in situations where there is a large number of features to be considered for each individual, the process of classification is rarely on an all-or-none basis; individuals are identified as belonging to the same group even though they are not identical in every particular.

Thus, with increasing numbers of variables, the process of classification is liable to become increasingly subjective. The computer has made it possible for large numbers of variables to be handled, and for individuals to be grouped according to degrees of similarity, in an entirely objective manner. This is the basis of numerical taxonomy, in which groups or clusters are formed, and the cluster analyses reported here illustrate the application of the technique to histological data.

Whilst computer-aided cluster analysis is an objective method of classification, the end result is no more objective than the data on which the analysis is based; that is the reason for the efforts made in this study to record histological findings without bias and without any attempt at interpretation. Also, it must be emphasized that, however good the mathematical technique, the clusters formed can be meaningful only if the data contained the necessary information: we are not necessarily aware of the features that will permit a clear distinction to be made between one entity and another.

Amongst the more successful applications of cluster analysis in the field of medical science have been the grouping or classification of micro-organisms (see, for example, Colman and Williams, 1967; Colman, 1968; Drucker and Melville, 1969), and the bibliography provided by Carlsson (1968) lists much of the earlier work in this field. The work of Baron and Fraser (1968) and Fraser and Baron (1968) provides examples of cluster analysis for diagnostic purposes: for a more general review of the use of the computer for diagnosis, see Boyle and Anderson (1968) and Anderson and Boyle (1968).

In our present study, we have compared the results of our own conventional, subjective methods of making a histological diagnosis, with the results of an objective computer analysis of the histological data. We wished to explore the possibility of making our methods less subjective, and of improving our diagnostic and prognostic criteria.

The analysis by subjectively selected criteria clearly shows, as we had expected, that a first attempt to state a few histological features that would lead to a diagnosis of lichen planus or " leukoplakia " was not successful. Such an approach is capable of refinement and improvement, particularly if the computer is used for a detailed analysis of the features that most clearly distinguish between one diagnostic group and another. Further studies on this type of discriminant analysis will be reported elsewhere.

The cluster analyses are also capable of refinement, but even in their present form they show an encouraging degree of separation of the diagnostic groups. In the present cluster analyses, the computer was provided with 41 variables for each case. However, each of these variables was given equal weight. Experience indicates that some of these variables are of particular value in distinguishing one disease from another, whilst other variables help little or not at all in making the distinction; such variables represent " noise " and might be better eliminated from the data to be analysed. Those that remain can be weighted according to their importance, and the degree of importance can be calculated rather than estimated.

It is important to have a clear view of the objectives of this type of computer-assisted study. Firstly, the computer can be used to facilitate the preparation of a large volume of simple data, such as the data on the frequency with which various tissue changes are found in each disease. These data could be obtained by other means; the computer only reduces the tedium of sorting many thousands of items.

Again at the level of a simple sorting process, the computer has enabled us to test the preliminary hypothesis regarding the tissue changes that lead us to a given diagnosis.

The cluster analyses require calculations of such complexity that it would be impractical for us to attempt them without the computer. These cluster analyses enable us to start testing the validity of our diagnostic groups, and the discriminant analyses to be reported later allow us to know, in quantitative terms, how different the groups are. Furthermore, these discriminant analyses will quantitate the importance of each histological variable, and this information can then be used to refine the methods of cluster analysis.

It is not suggested that the computer can take over the work of the histopathologist (although the time may come when this happens, in certain aspects of our work). But the computer can already help us to make our work less subjective, and it may draw to our attention items of diagnostic or prognostic importance that we have not yet realized to be significant.

In the most recent parts of our study, we have reached the stage where the computer suggests that some of our cases have been wrongly diagnosed; when those cases are reviewed, it is sometimes clear that the computer is right.

We gratefully acknowledge the financial support given by the British Empire Cancer Campaign for Research, and the advice given by Miss Ann Russel of the Institute of Computer Science, University of London.

We are indebted to Mr. Michael Clarke, not only for the cluster analyses and the Appendix on the method used, but also for advice on other aspects of this computer analysis.

### REFERENCES

Anderson, J. A. and Boyle, J. A.—(1968) *Br. med. Bull.*, **24,** 230.
Andreasen, J. O.—(1968) *Oral surg.*, **25,** 31.
Baron, D. N. and Fraser, P. M.—(1968) *Br. med. Bull.*, **24,** 236.
Boyle, J. A. and Anderson, J. A.—(1968) *Br. med. Bull.*, **24,** 224.
Cahn, L. R., Eisenbud, L. and Blake, M. N.—(1961) *Oral surg.*, **14,** 596.—(1962) *Oral surg.*, **15,** 458.
Carlsson, J.—(1968) *Odont. Revy.*, **19,** 137.
Colman, G.—(1968) *J. gen. Microbiol.*, **50,** 149.
Colman, G. and Williams, R. E. O.—(1967) *Int. J. syst. Bact.*, **17,** 306.
Cooke, B. E. D.—(1954) *Br. dent. J.*, **96,** 1.
Darling, A. I. and Crabb, H. S. M.—(1954) *Oral surg.*, **7,** 1276.
Doyle, J. L., Manhold, J. H. and Weisinger, E.—(1968) *Oral surg.*, **26,** 667.
Drucker, D. B. and Melville, T. H.—(1969) *Nature, Lond.*, **221,** 664.
Fraser, P. M. and Baron, D. N.—(1968) *Proc. R. Soc. Med.*, **61,** 1043.
Komori, A., Welton, W. A. and Kelln, E. E.—(1966) *Oral surg.*, **22,** 752.
Kramer, I. R. H.—(1969) *Ann. R. Coll. Surg.*, **45,** 340.
Kramer, I. R. H., Lucas, R. B., El-Labban, N. and Lister, L.—(1969) *J. dent. Res.*, **48,** 1096.
Levin, H. L.—(1957) *U.S. arm. Forces med. J.*, **8,** 198.

Pindborg, J. J. Renstrup, G., Jølst, O. and Roed-Petersen, B.—(1968) *J. Am. dent. Ass.*, **76**, 767.
Radden, B. G.—(1966) *Med. J. Aust.*, **1**, 441.

### APPENDIX ON CLUSTER ANALYSIS

M. R. B. Clarke
*From the Institute of Computer Science, University of London*

Suppose that we have $N$ individuals on each of whom $p$, possibly correlated, measurements have been made.

It would be of interest to have a technique for dividing the data into groups solely on the basis of the $p$ measurements for each individual. By comparing this grouping with the diagnostic groups made by the pathologist we would hope to gain some insight into the diagnostic process, and also perhaps have a useful screening technique. This procedure has come to be known as cluster analysis and it is only recently that any interest has been shown in it.

Cluster analysis cannot be said to have reached an advanced stage of development and, as yet, there is no generally accepted method. This is hardly surprising, perhaps, because the way in which clusters are formed must depend on what underlying model is assumed for the data. Any particular method will only be optimal with respect to classification errors for a certain type of data, although one might hope that some methods will be nearly optimal in a wide variety of commonly occurring situations. It is suggested that the method used here has this property and some general reasons for thinking so are given at the end, although no theoretical results have yet been found.

There are three aspects of any clustering method:

(a) A criterion of good clustering.
(b) An algorithm for sorting the data into clusters in such a way as to maximize this criterion.
(c) A method of determining how many significant clusters there are in the data.

The criterion proposed here is defined in terms of an inter-subject distance which itself is a metric imposed on the $p$-dimensional measurement space. Let $d_i$ be the distance between individuals $i$ and $j$ and suppose we have a grouping of $N$ individuals into $k$ clusters such that there are $n_j$ ($>1$) in cluster $j$, $\sum_{j=1}^{k} n_j = N$. Then we define the criterion $C$ as the average between-cluster distance divided by the average within-cluster distance.

$$C = \frac{m(T-W)}{(M-m)W} \text{ where } m = \sum_{j=1}^{k} \tfrac{1}{2}n_j(n_j - 1),$$

where $M = \frac{1}{2}N(N-1)$ and $W = \Sigma d_{ij}$ over all $ij$ in the same cluster and $T = \Sigma d_{ij}$ over all $i$ and $j$. $M$ and $T$ are constant with respect to all clusterings, $M$ and $W$ vary. The algorithm for maximizing this with respect to cluster allocation is of the steepest ascent variety. For a given clustering an $N \times (k-1)$ matrix is stored, giving the improvement in criterion that would result from moving an individual to any of the other clusters. The procedure is thus iterative; at each stage the largest improvement (element of this matrix) is selected. The corresponding individual is moved to the indicated group, the elements of the matrix recalculated and the procedure repeated until no positive element remains in the matrix, indicating that a maximum has been reached. Not every element of the matrix has to be recalculated, but only those $2N$ of them involving the group that was moved from and the group that was moved to. Suppose these are $r$ and $s$ respectively, and that the sums of distances of the element moved ($i$) from the other elements of group $r$ and $s$ are $E_{ir}$ and $E_{is}$. Then the new value of $m$ is

$$m' = m + n_s - n_r + 1$$

and of $W$

$$W' = W + E_{is} - E_{ir}$$

and the new value of the criterion can be calculated from these. In practice the iterations are extremely rapid.

To start the iteration off, a first approximation is needed. One method used to obtain this was first to take the two elements furthest apart and put these in different clusters, then to take the element furthest away from both these and put this in another cluster, and so on until $k$ elements have been taken. These then form the nuclei of $k$ clusters and the remaining ($N$–$k$) individuals are put into the cluster in which they lie nearest to the nucleus. This then serves as an initial approximation for the iteration. In practice, although this gives such a good clustering that very few iterations are required (typically about 0·05 $N$) it is extremely time-consuming and a quicker method is to allocate elements to clusters at random initially. About 0·75 $N$ iterations are then required, but for large $N$ this can be substantially quicker. This raises the question as to whether the same solution is always attained from different starting points, or are there local maxima of the criterion? It is possible to demonstrate a local maximum in a very abnormal situation, but in extensive experiments with simulated realistic data and artificially bad starting points none has been found in practice.

The criterion and algorithm described above are independent of the metric and this can than be chosen to fit the kind of measurements that have been made. For a multivariate normal model the Mahalanobis distance $(x-y)'\Sigma^{-1}(x-y)$ can be used where $\Sigma$ is the dispersion matrix and $x$ and $y$ are sample vectors. If $\Sigma$ is the identity matrix then this is a Euclidean distance and it can easily be shown that the criterion becomes the between-cluster variance divided by the within-cluster variance. In the present application a simpler distance measure was used. The data was dichotomized into binary variables that were coded zero or unity, and the distance between two individuals was the number of zeros or ones that they did not have in common. In other words, the two binary vectors were non-equivalenced and the number of ones was counted. This method is recommended with a lot of data where store is short because the binary digits can be packed many to a computer word, and the distances are not stored, ($\frac{1}{2}N(N-1)$ often being a large number) but computed each time they are required, this being an extremely fast operation.

The method was extensively tested on simulated binary data before being used for the present study, in an attempt to discover how many noise variables would obscure the underlying clustering. It was found that with 20 classifying variables, where every individual in the same group had the same pattern, at least 400 random binary digits could be added to these data before the clustering was spoilt. In other words, the clustering was reproduced if only one in twenty of the $p$ measurements was a genuine diagnostic indicator. Naturally these results are not conclusive but serve to illustrate the power of the method.

It will be noted that the criterion of average between-group distance divided by within-group distance is very similar to the discriminant function criterion and, since discriminant analysis is known to be robust against departures from distribution assumptions, it is this that leads us to claim a degree of optimality for the present method, in a wide variety of situations. It is clearly extremely difficult to get the exact distribution of the maximized criterion for any particular parent distribution, as it involves order statistics.

References are given at the end of this Appendix to some other methods of cluster analysis. Some of these are what have come to be called hierarchial methods, in which clusters divide and sub-divide rather than all starting on an equal footing. The present method can be used for this if only 2 clusters are specified at each stage.

### REFERENCES

Bonner, R. E.—(1964) *IBM Jl Res. Dev.*, **8,** 22.

Jardine, N. and Gibson, R.—(1968) *Comput. J.*, **11,** 177.

Lance, G. N. and Williams, W. T.—(1967) *Comput. J.*, **9,** 373.—(1967) *Comput. J.*, **10,** 271.—(1968) *Comput., J.*, **11,** 195.

Sokol, R. R. and Sneath, P. H. A.—(1963) ‘ Numerical Taxonomy ’. London (W. H. Freeman).


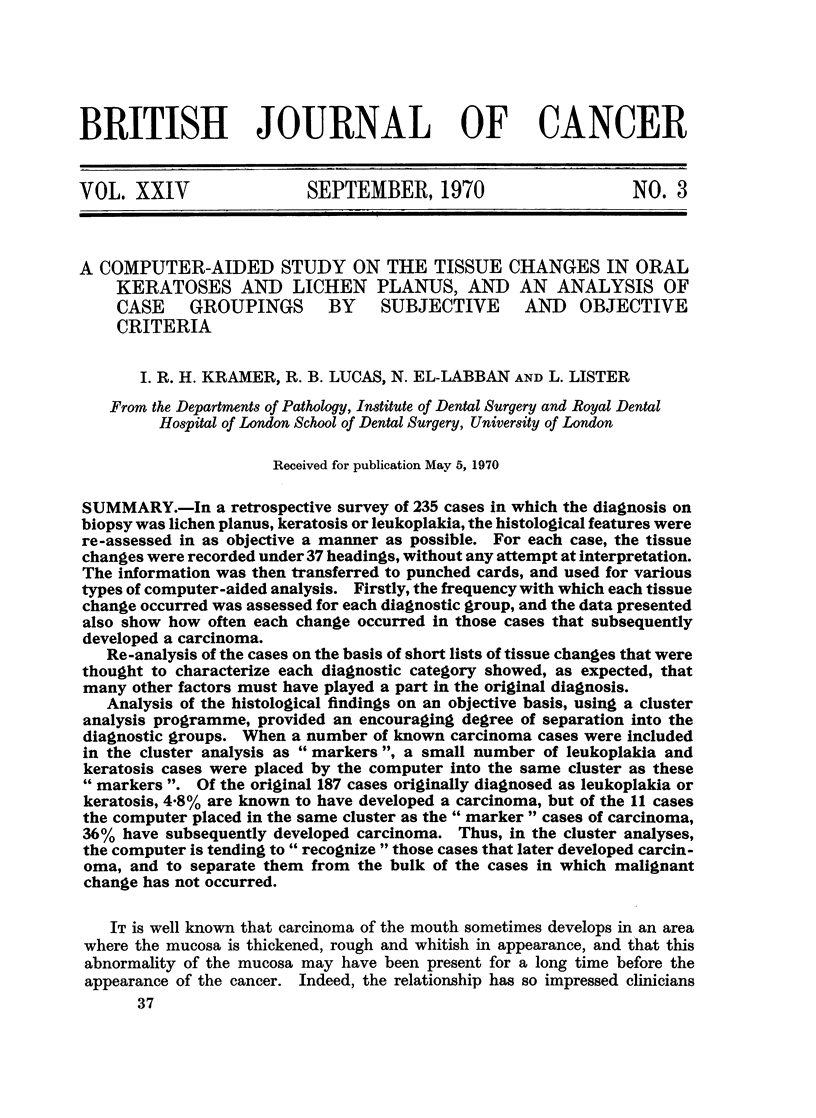

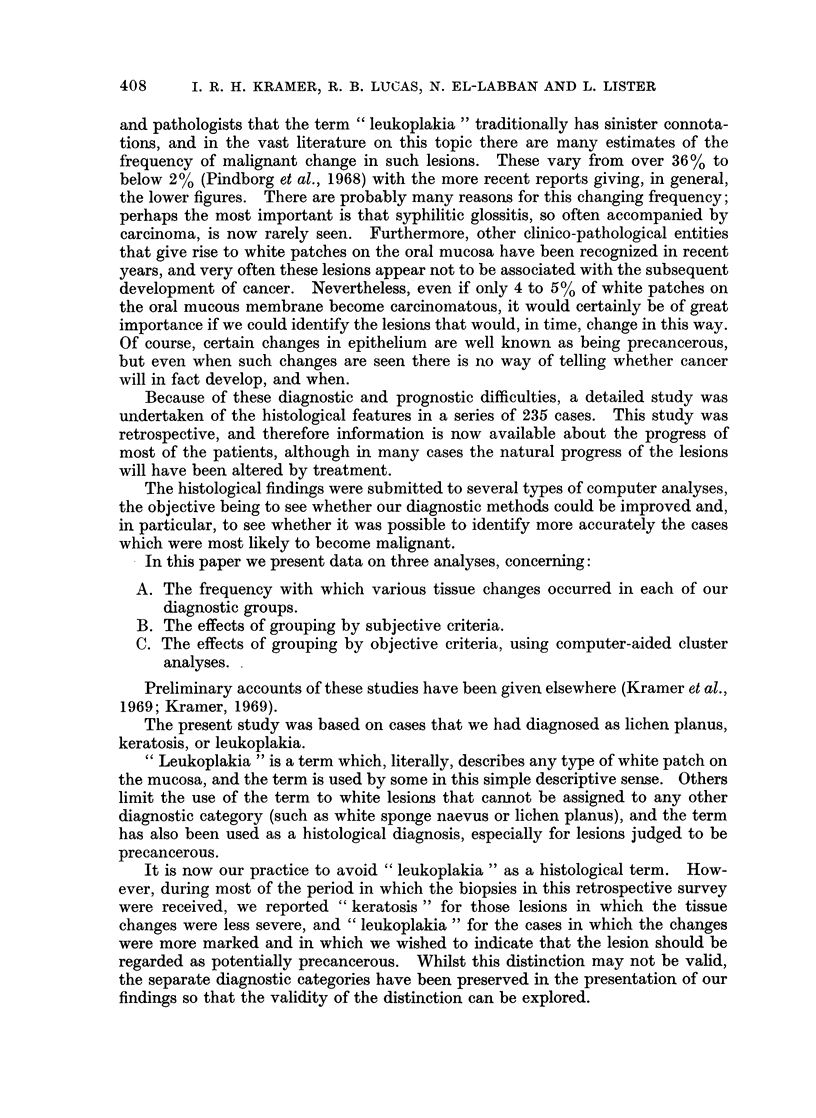

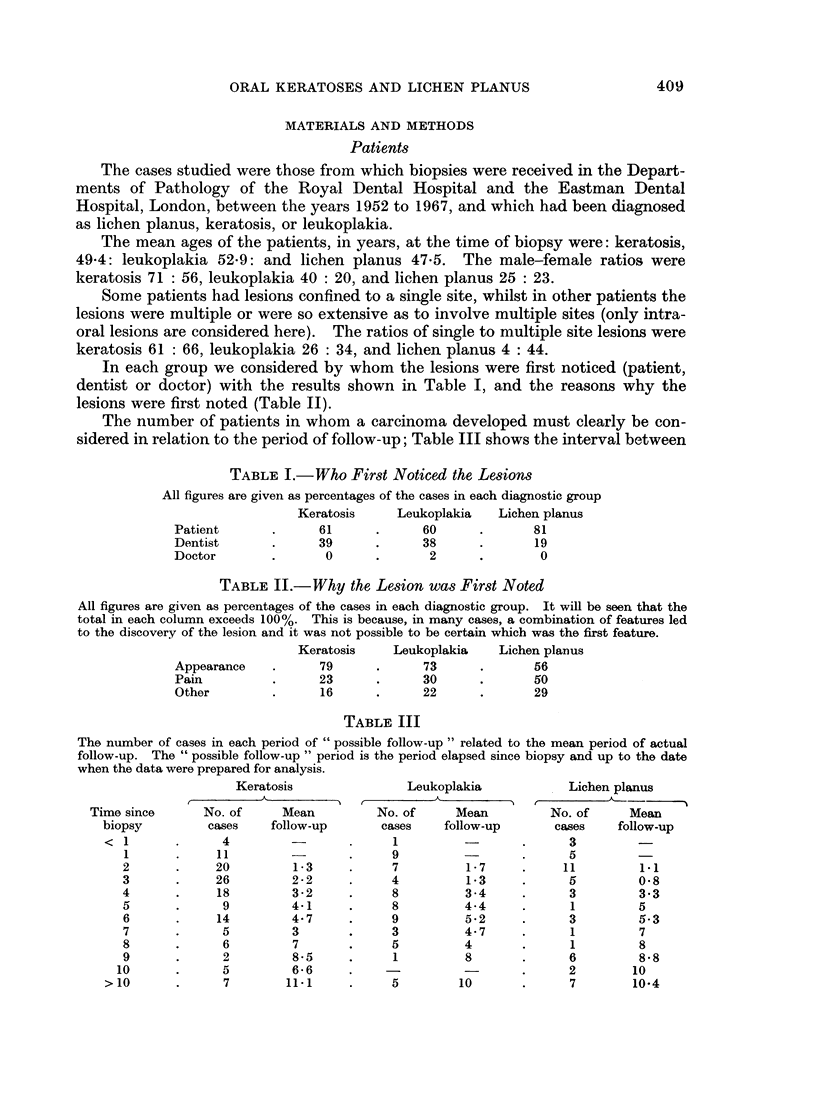

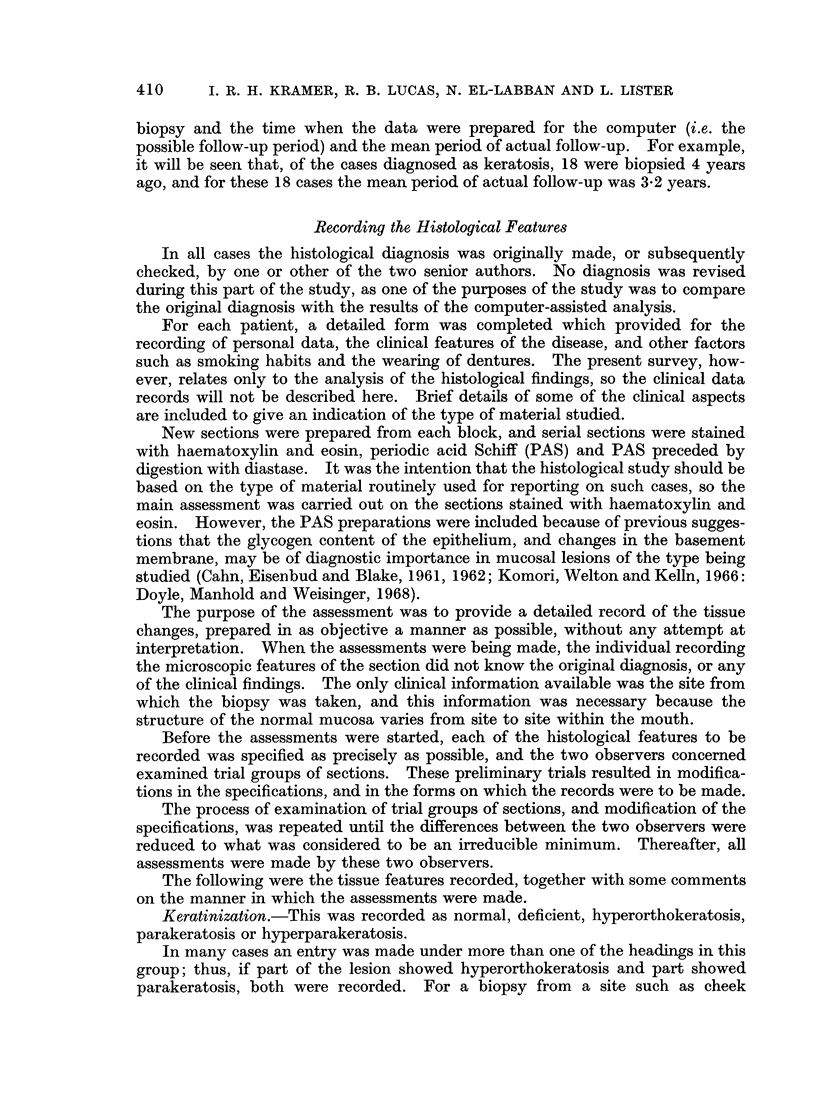

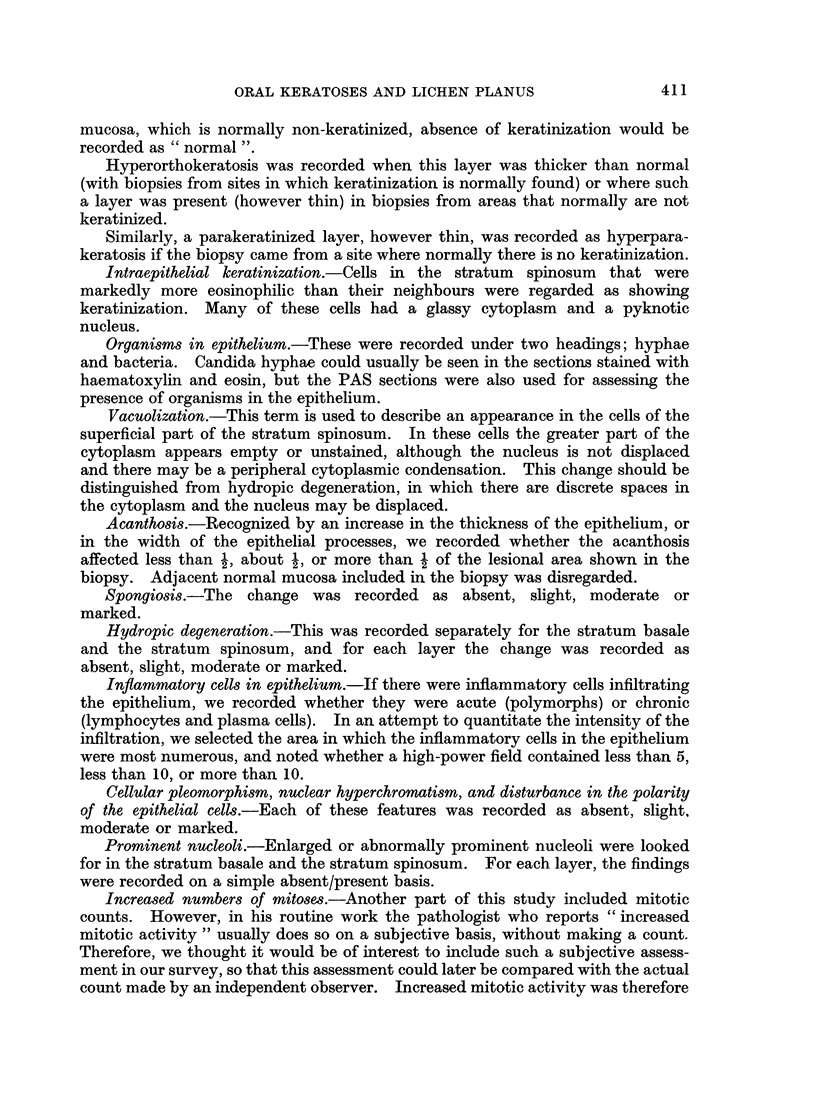

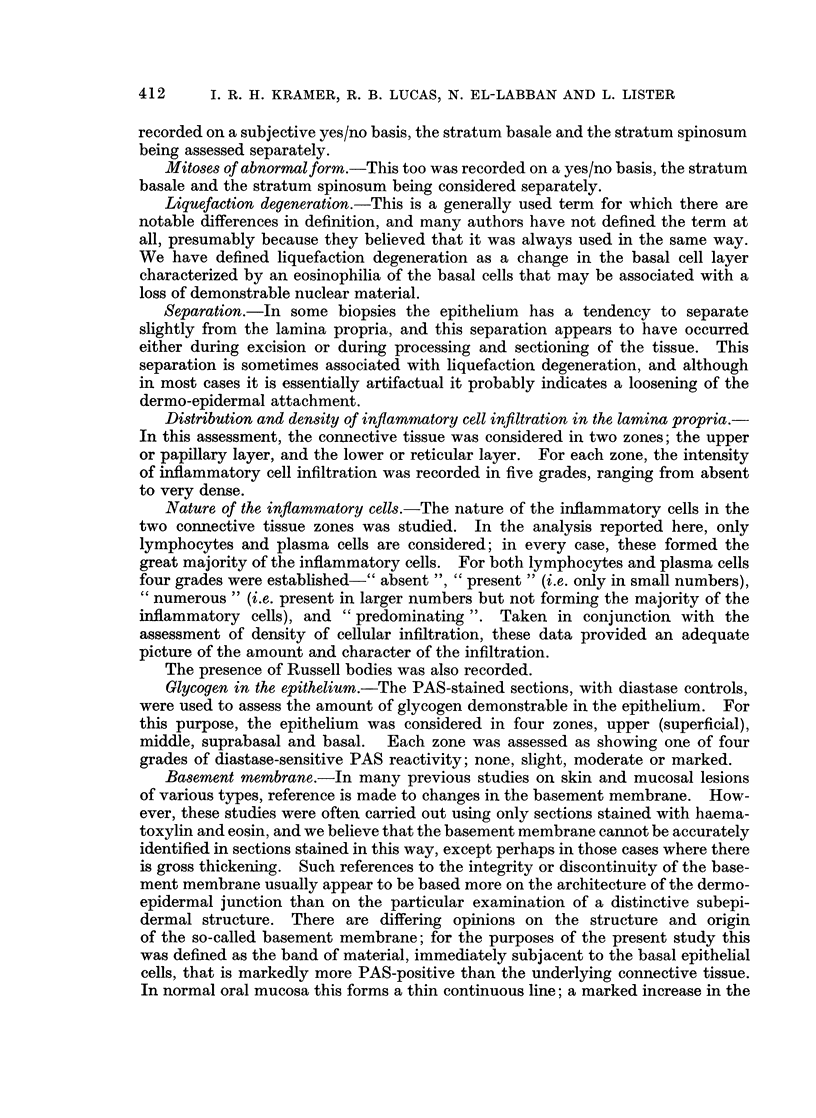

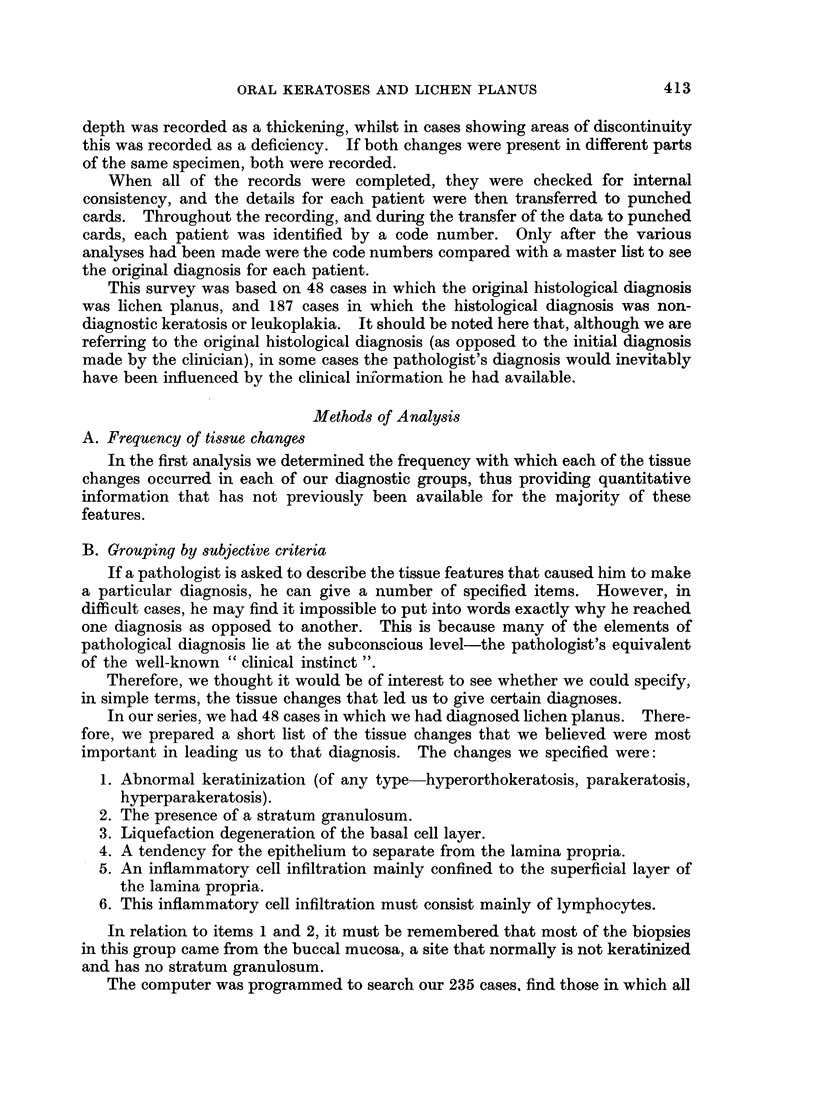

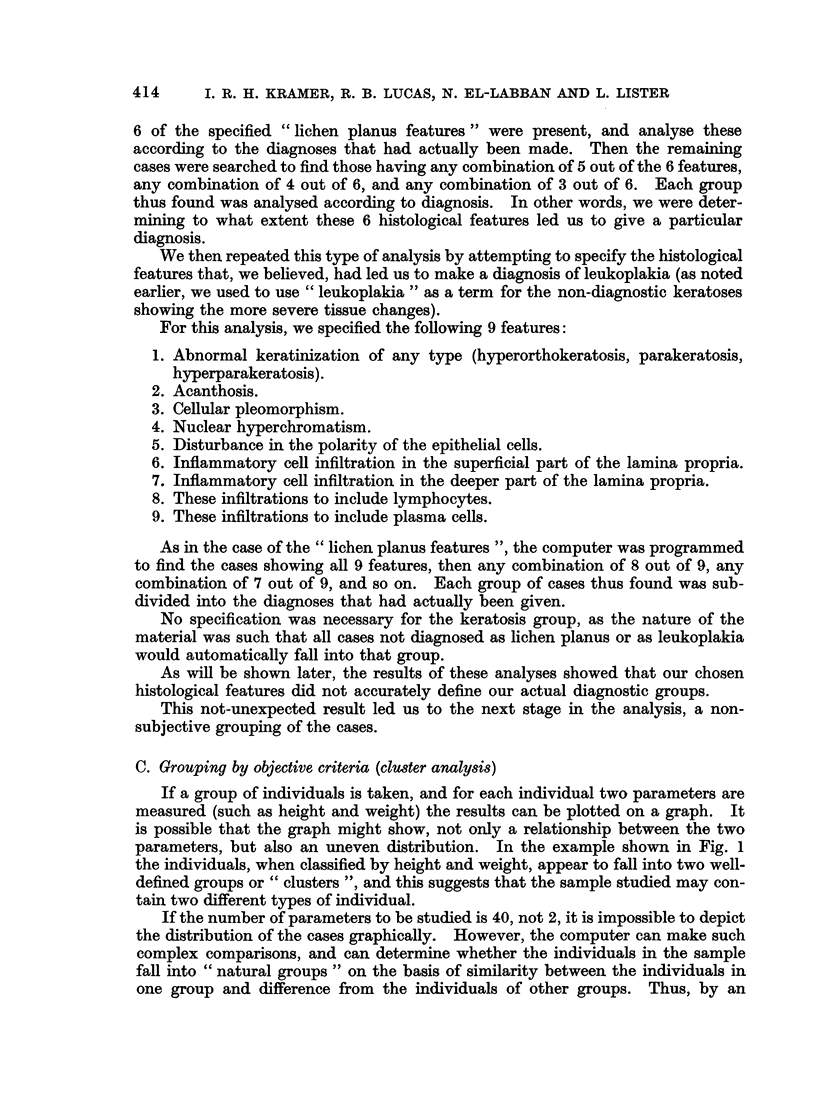

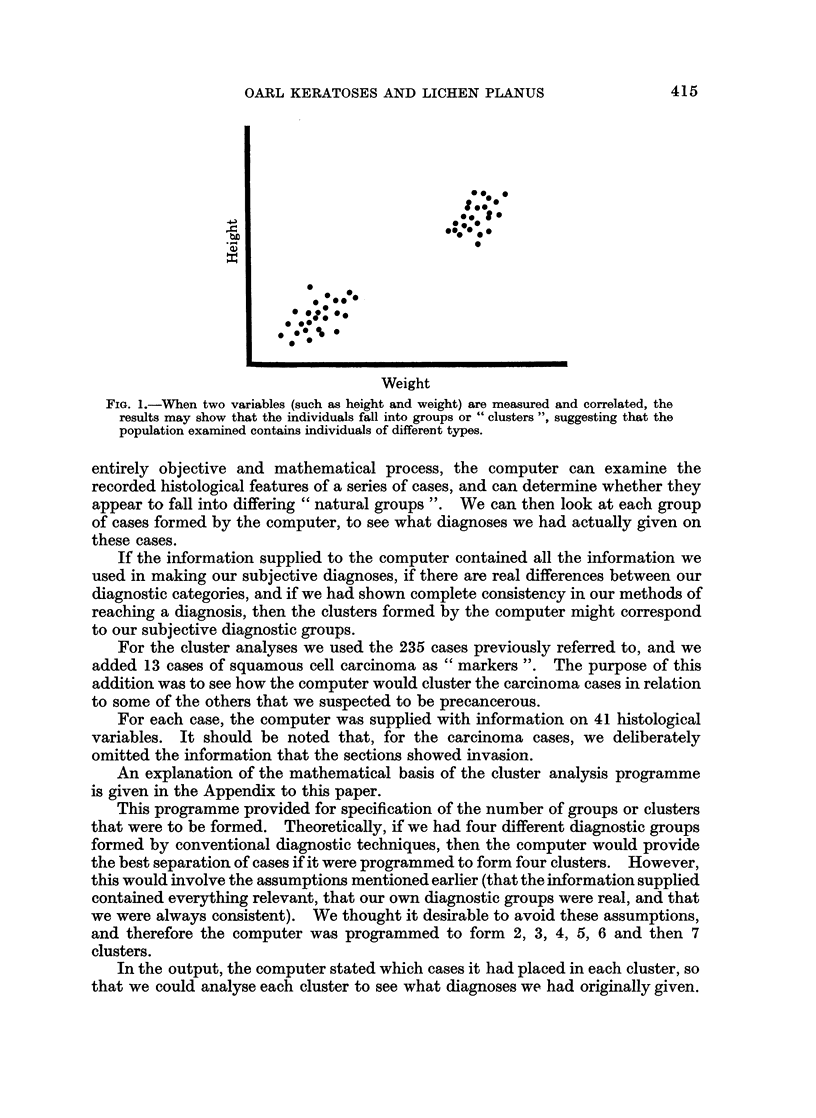

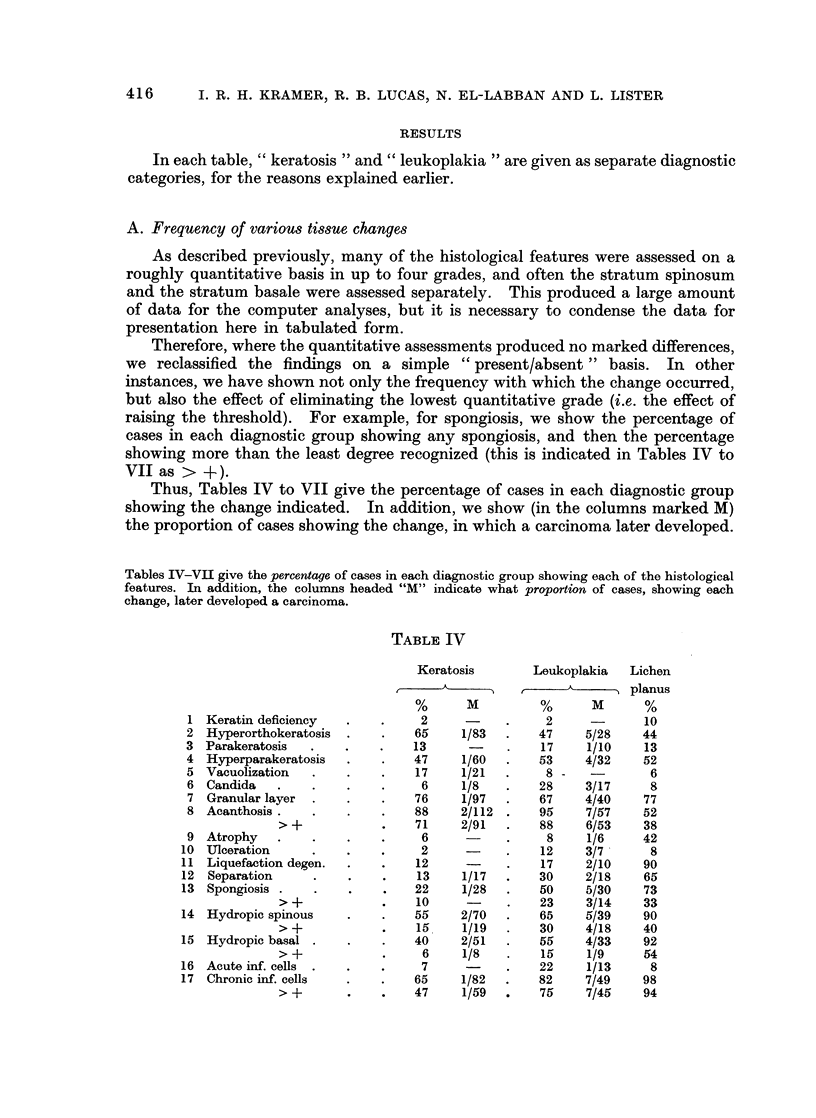

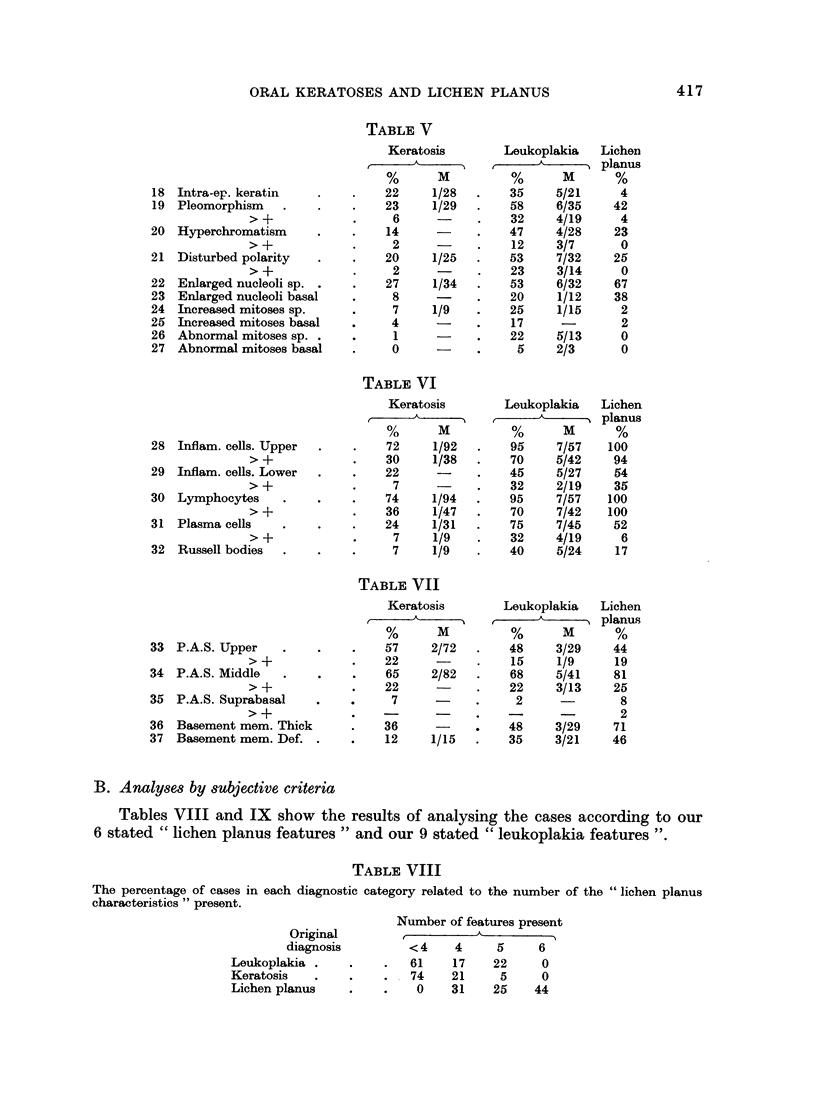

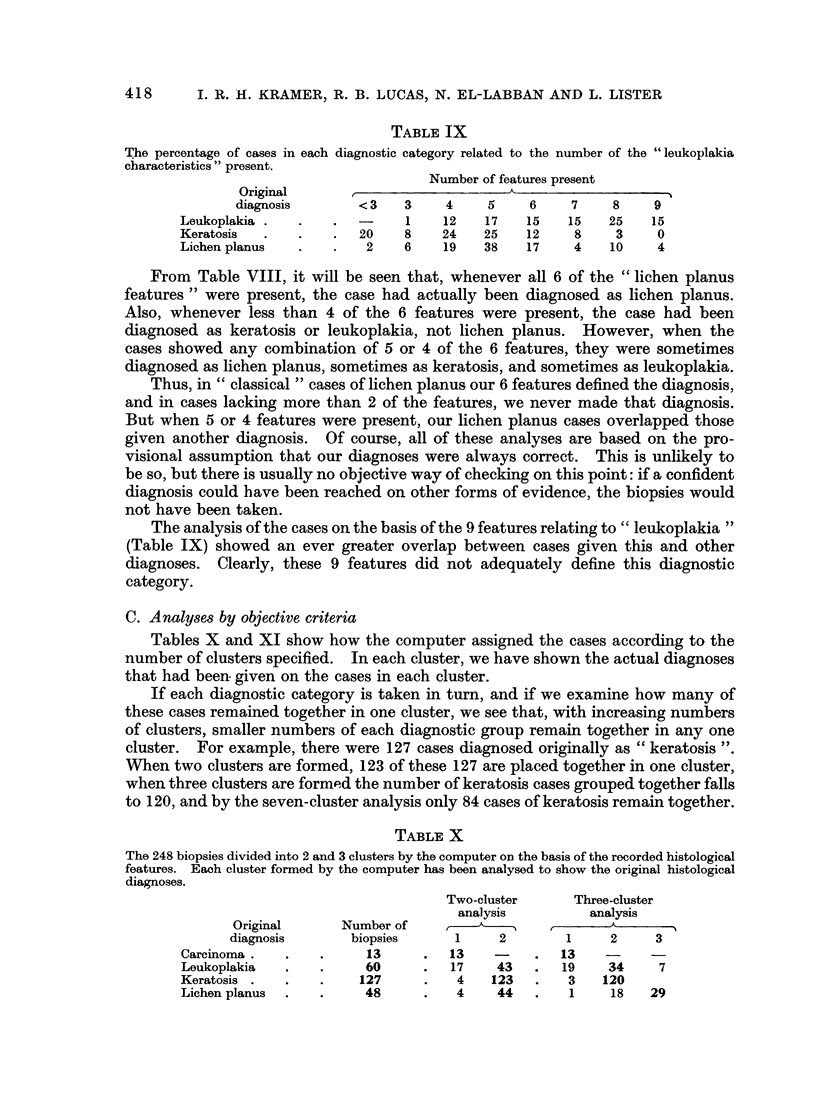

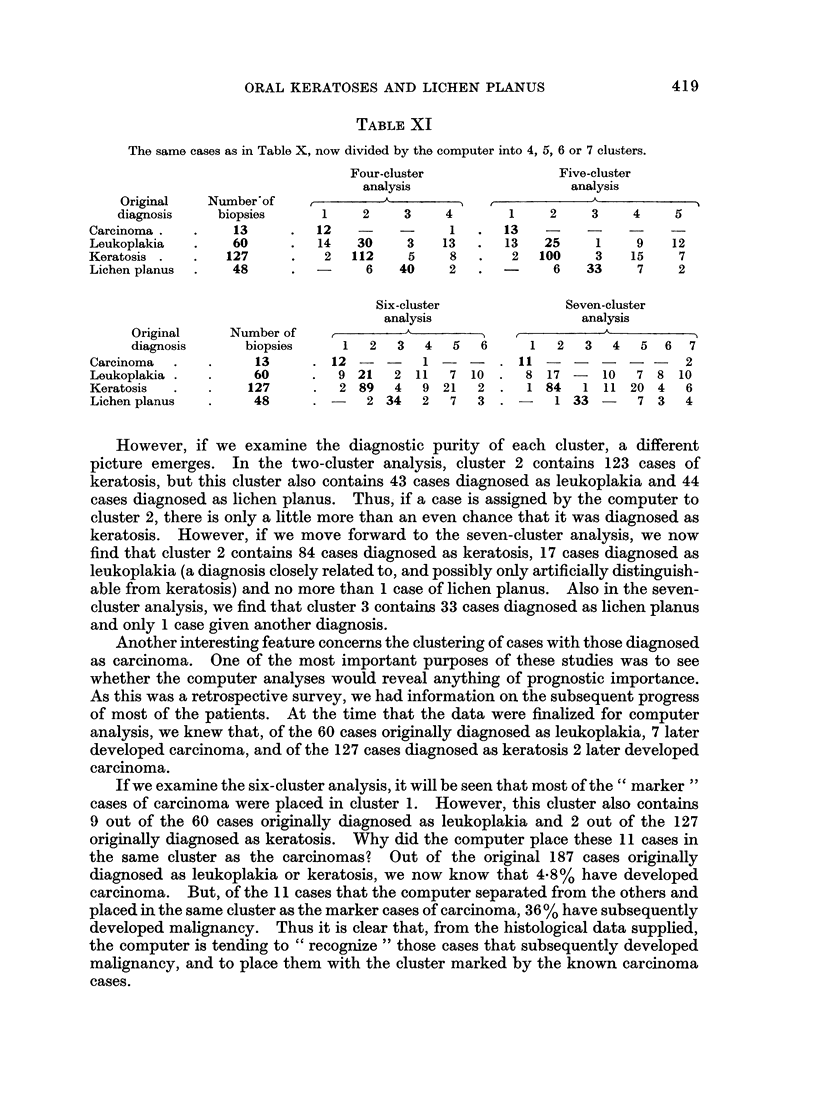

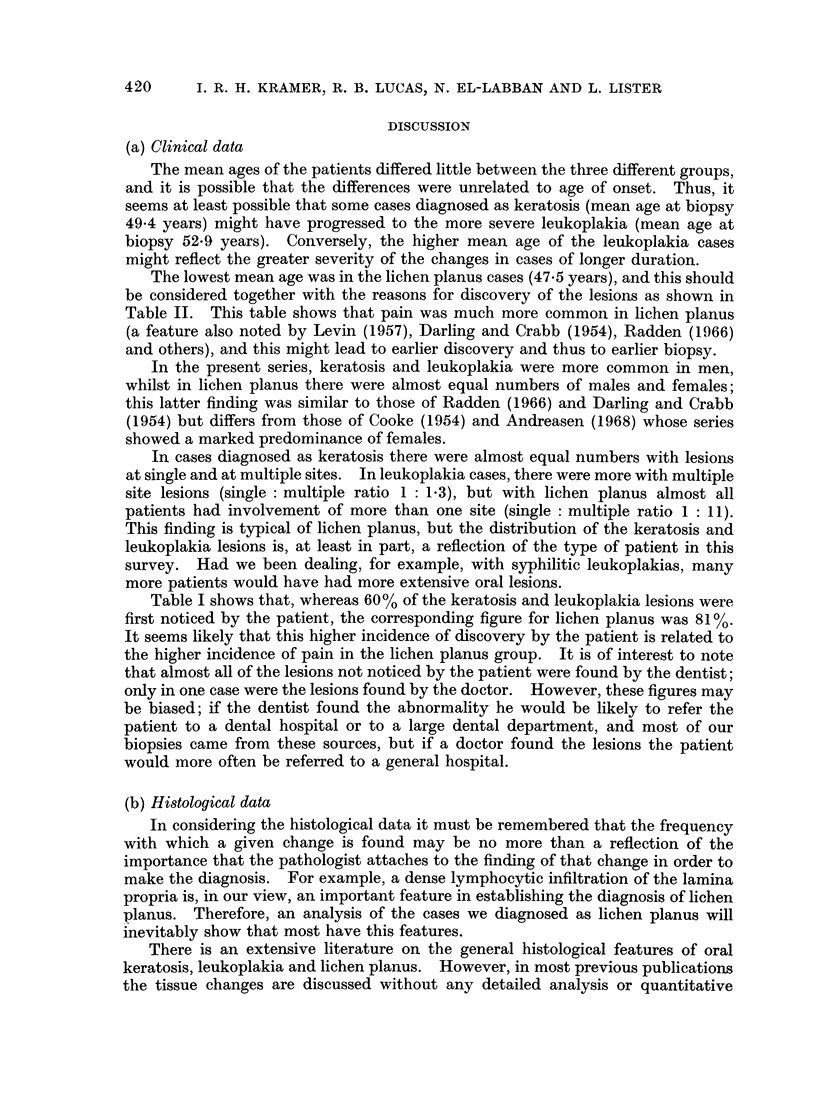

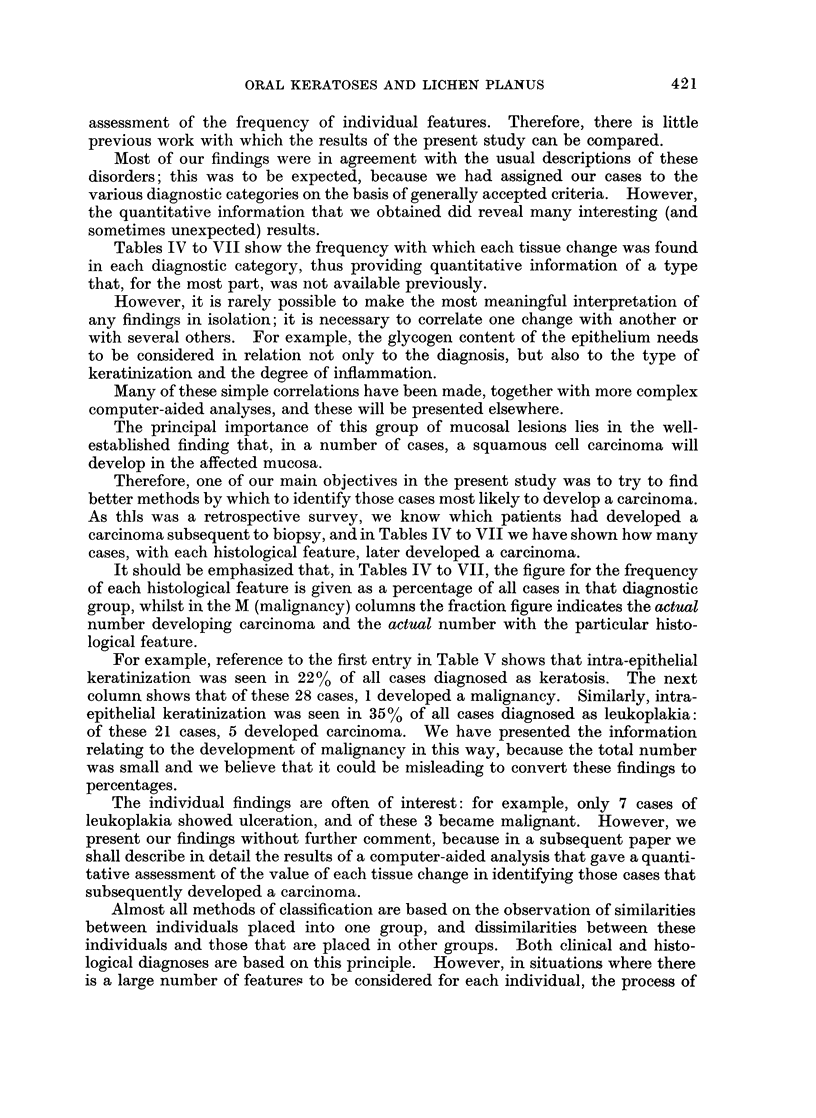

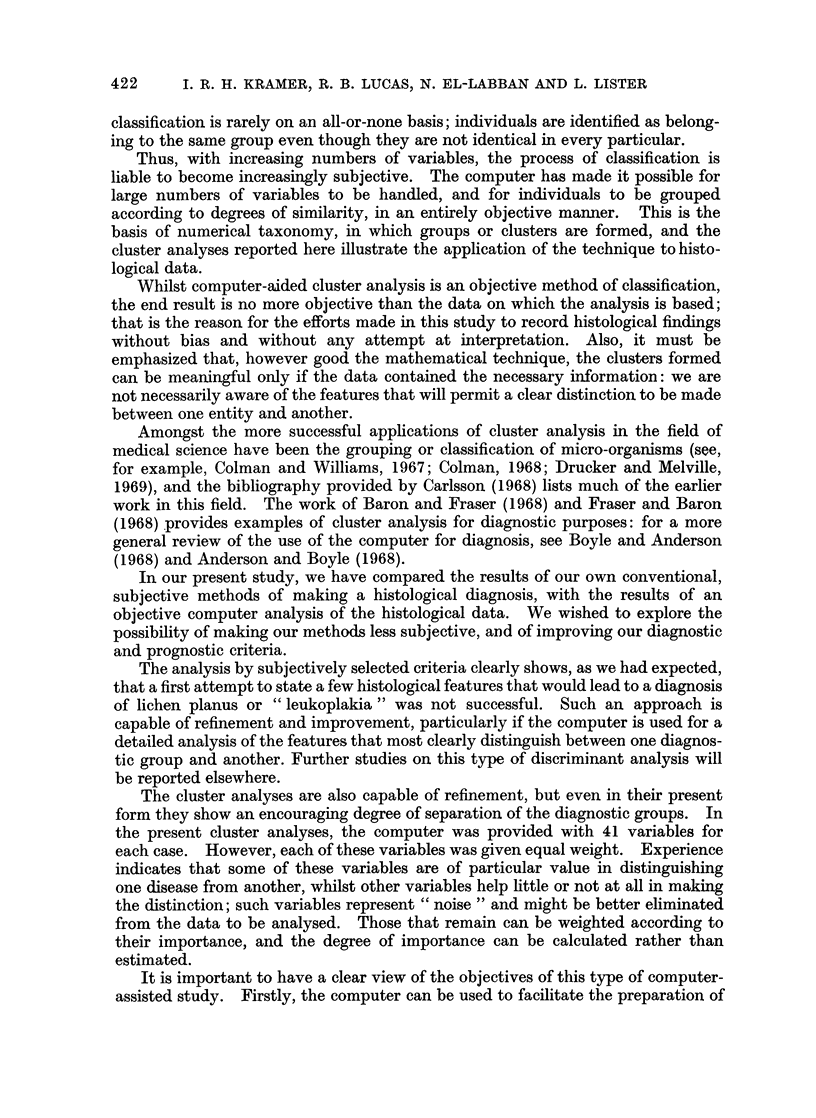

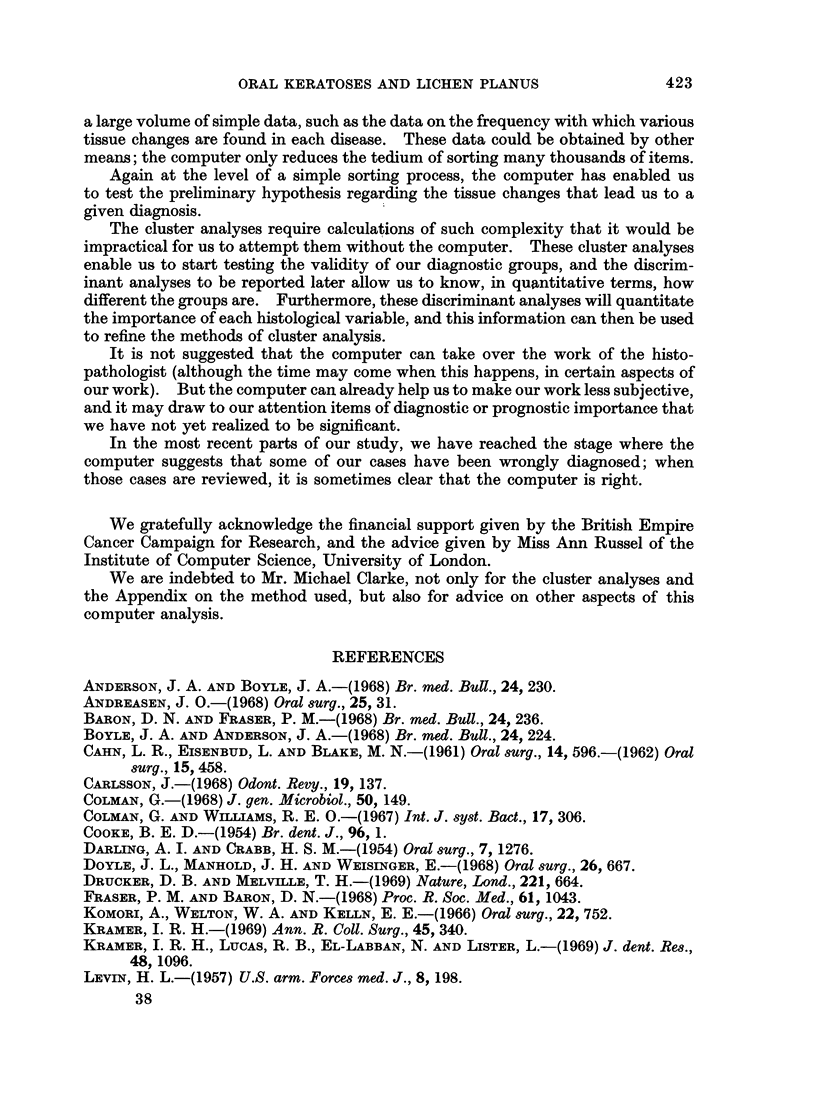

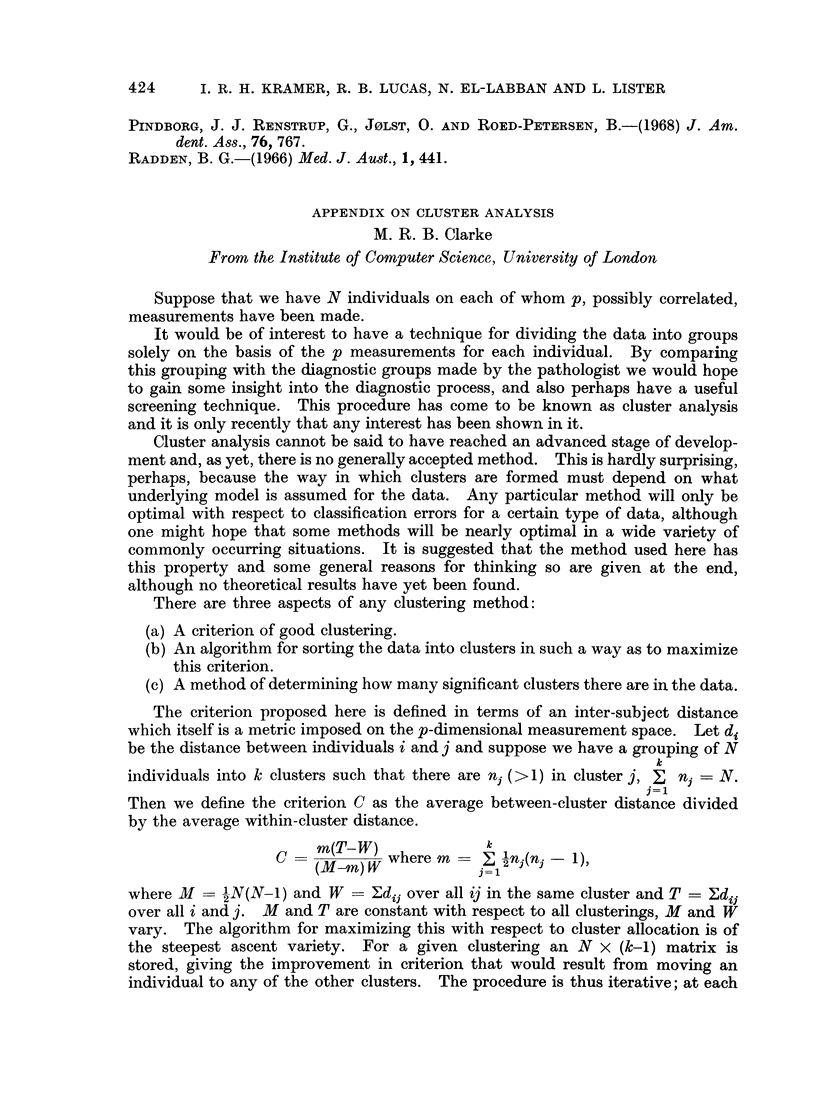

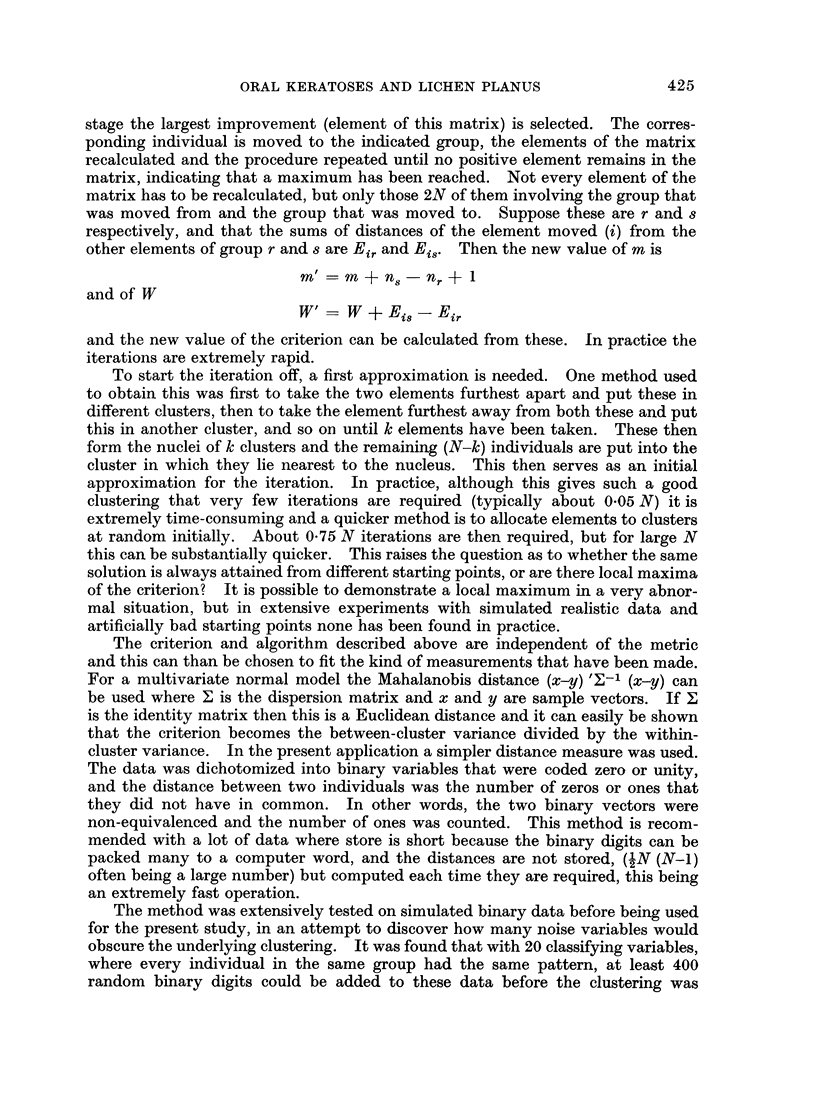

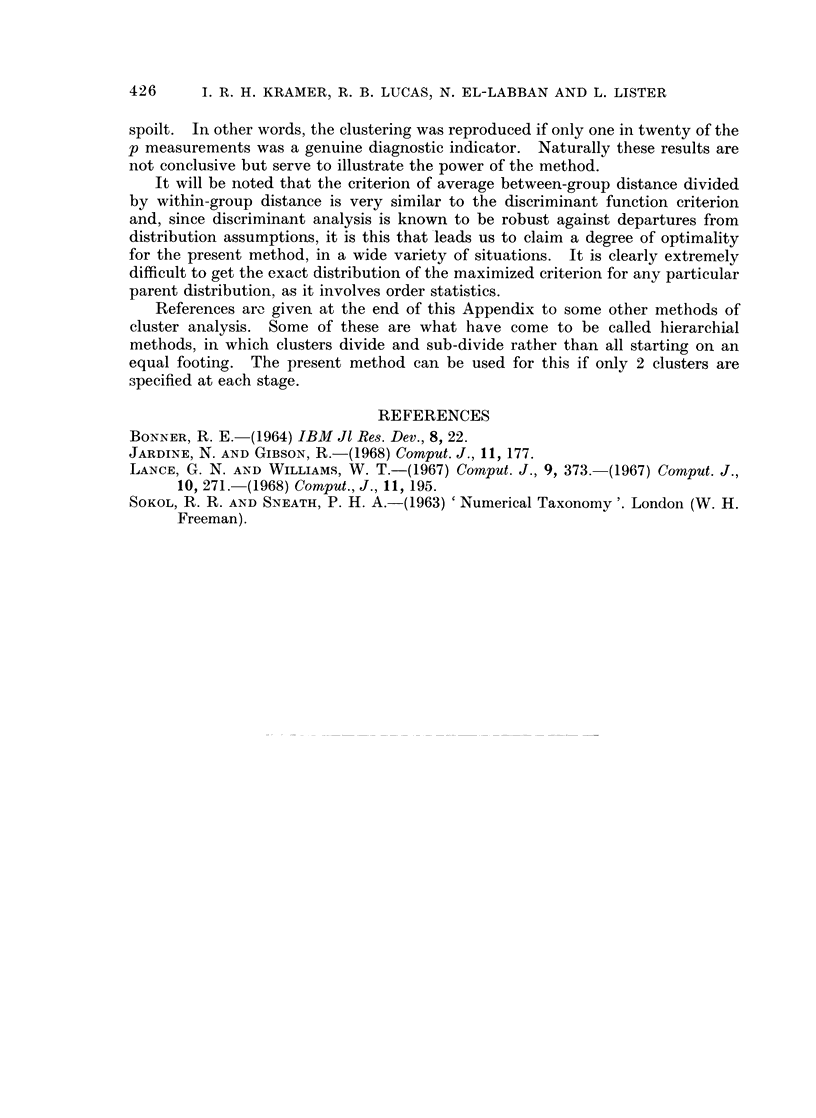


## References

[OCR_01075] Anderson J. A., Boyle J. A. (1968). Computer diagnosis: statistical aspects.. Br Med Bull.

[OCR_01083] CAHN L. R., EISENBUD L., BLAKE M. N. (1961). Histochemical analyses of white lesions of the mouth. I. The basement membrane.. Oral Surg Oral Med Oral Pathol.

[OCR_01085] Carlsson J. (1968). A numerical taxonomic study of human oral streptococci.. Odontol Revy.

[OCR_01087] Colman G. (1968). The application of computers to the classification of streptococci.. J Gen Microbiol.

[OCR_01095] Drucker D. B., Melville T. H. (1969). Computer classification of streptococci, mostly of oral origin.. Nature.

[OCR_01099] Kramer I. R. (1969). Precancerous conditions of the oral mucosa. A computer-aided study.. Ann R Coll Surg Engl.

[OCR_01105] LEVIN H. L. (1957). Oral lichen planus and leukoplakia; differential diagnosis and treatment.. U S Armed Forces Med J.

[OCR_01113] Pindborg J. J., Jolst O., Renstrup G., Roed-Petersen B. (1968). Studies in oral leukoplakia: a preliminary report on the period pervalence of malignant transformation in leukoplakia based on a follow-up study of 248 patients.. J Am Dent Assoc.

[OCR_01257] Radden B. G., Reade P. C. (1966). Oral lichen planus.. Med J Aust.

